# Influence of co-blending fly ash and ceramic waste powder on the performance and microstructure of cementitious substrates under sulfate dry-wet cycle attack

**DOI:** 10.1371/journal.pone.0314277

**Published:** 2025-02-21

**Authors:** Xinyan Wang, Lu Zhang, Haizhou Li

**Affiliations:** 1 School of Civil Engineering and Geomatics, Shandong University of Technology, Zibo, China; 2 Key Laboratory for Liquid-Solid Structural Evolution & Processing of Materials of Ministry of Education, Shandong University, Jinan, China; 3 School of Resources and Environmental Engineering, Shandong University of Technology, Zibo, China; Mirpur University of Science and Technology, PAKISTAN

## Abstract

This study examines the properties of cement-based materials incorporating composite additions of fly ash and ceramic waste powder (CWP) as supplementary cementitious materials (SCM). The resistance of the materials to sulfate erosion under dry-wet cycling conditions was investigated through experimental testing. A Box-Behnken Design was employed to establish a model using three factors: the replacement ratio of cement by SCMs, the mass ratio of CWP to SCMs, and the water-to-binder ratio. The response variable was the mass loss rate due to sulfate erosion after 24 cycles of dry-wet cycling. Significance analysis of single-factor and multiple-factor interactions was conducted based on the response surface model. The research findings indicate that the cement-based materials with combined additions of fly ash and CWP exhibit optimal resistance to sulfate erosion under dry-wet cycling conditions. The water-to-binder ratio was identified as the most significant factor affecting the corrosion resistance of the cement-based materials at 7 days of curing. The dosage of ceramic waste powder influenced the corrosion performance of the cement-based materials at 28 days of curing. The content of SCMs affected the corrosion resistance of the cement-based materials after 56 days of curing. Comparative analysis of the grayscale three-dimensional distribution map and histogram of the cement-based materials with SCMs revealed an increase in the compactness of the matrix.

## 1. Introduction

The development of structural building materials is significantly constrained by various factors, including resources, energy, and environmental considerations [1]. Cement plays a crucial role as a major component of structural building materials, and the excessive production and consumption of cement have led to increasingly pressing energy issues. The cement production process consumes a substantial amount of resources such as standard coal, limestone, and clay. Approximately 180 kilograms of standard coal are consumed per ton of cement clinker. Combustion in the production process releases harmful gases, including CO_2_ and sulfur oxides, which contribute to the greenhouse effect and the formation of acid rain, thus exacerbating the environmental burden on the Earth [2]. According to statistics, the cement industry accounts for one-tenth of the world’s CO_2_ emissions, amounting to 10 billion tons. It is anticipated that this figure will rise to one-fourth by 2050 [3]. The CO_2_ emissions from the cement industry have significant implications for the global environment. Therefore, restraining cement consumption serves as a key indicator for achieving carbon peak and carbon neutrality [4].

The utilization of supplementary cementitious material (SCM) as a partial substitutes for cement is an effective measure to mitigate carbon emissions associated with cement production. This approach not only protects the environment but also significantly reduces the consumption of natural resources. Current research primarily focuses on the utilization of fly ash [5], silica fume [6], and slag powder [7] to prepare cement-based construction materials [8]. By reducing the cement content, it is possible to decrease the production costs of precast materials [9], while simultaneously improving the workability and durability of cement-based materials [10]. However, due to the increasing promotion of clean energy, the capacity and proportion of natural gas power generation have been expanding, leading to significant limitations on coal-fired power plants, which are the primary source of fly ash. As a result, the availability of fly ash has become insufficient. Additionally, strict limitations on the increase in steel production capacity have resulted in a reduction in the production of slag powder, a byproduct of blast furnace steelmaking [11]. These factors have collectively led to a scarcity of SCM and subsequent price increases. Therefore, it is imperative to research and develop new types of SCM.

Ceramic waste powder (CWP) shares similar characteristics with traditional supplementary materials, leading to the emergence of its use as an auxiliary cementitious material [12]. Ceramic tiles are produced by high-temperature sintering of clay with materials such as quartz or feldspar at 1200 degrees Celsius. CWP is a fine solid powder generated during the manufacturing processes of ceramic tiles, including shaping, cutting, grinding, and polishing. The main chemical components of CWP are Al_2_O_3_ and SiO_2_, exhibiting potential reactivity as a cementitious material in cement paste with properties similar to fly ash [13]. China boasts abundant reserves of ceramic clay and is the world’s largest producer of ceramic tiles, accounting for over two-thirds of the global annual production [14]. CWP is characterized by stable physical and chemical properties, poor degradability, and currently lacks effective recycling methods [15]. Simple landfilling or open-air stacking of CWP exacerbates the content of inhalable particulate matter in the environment, while burying it leads to long-term land occupation and pollution, posing a serious threat to water environments [16, 17]. The adverse ecological impact of CWP highlights the escalating conflict between the ceramic tile manufacturing industry and the ecological environment [18]. Developing CWP as a new auxiliary cementitious material to replace cement not only meets environmental requirements but also addresses resource challenges [19].

Research results have shown that ceramics have strong corrosion resistance, and their elements do not migrate to the substrate [20]. Matrix materials prepared by replacing cement with CWP up to 20% demonstrate significant advantages in terms of mechanical performance and durability [21]. By replacing cement with CWP up to 40%, the self-shrinkage of the cementitious matrix can be effectively reduced, and the toughness and stability of the matrix can be increased [22]. The rational combination of particle size distribution of various SCMs such as fly ash, silica fume, slag, and CWP can form a three-dimensional Si-O-Al rigid bonding structure [23]. It can refine the pore structure of the cement-based matrix, thereby effectively enhancing the material’s durability [24].

Sulfate erosion is a crucial issue that affects the durability of cement-based materials. The combined effect of temperature differences between day-night and sulfate erosion accelerates the rate of deterioration of cement-based materials [25]. During the process of sulfate erosion of cement-based materials, sulfates react with the hydration products of cement, specifically calcium hydroxide, resulting in a decrease in the pH value within the material and a reduction in the stability of the hydrates, ultimately leading to structural damage [26]. Additionally, corrosion generates expansive substances. If the expansive force exceeds the tensile strength of the base material, it will cause cracking in the cement matrix, significantly shortening the service life of cement-based materials [27]. The use of SCM to replace cement can effectively reduce the content of cementitious materials and consume excess expansive hydration products, thereby enhancing the resistance of the base material to sulfate erosion [28].

The response surface methodology is an experimental design method that combines mathematics and statistics. By establishing suitable mathematical models based on experimental data, it analyzes the influence of various factors on response values to determine the optimal process parameters under the combined influence of multiple factors. Response surface analysis encompasses experimental design, mathematical modeling, model analysis, and parameter optimization. Compared to orthogonal experiments, response surface methodology requires fewer experiments, offers higher precision, and can utilize imaging techniques to identify optimal conditions in experimental designs. The application of response surface methodology in optimizing cement-based material mixtures has garnered significant attention from scholars. For instance, Marzouki focused on the 14-day compressive strength of polymer-based sediment from Tunisian dams to ascertain the optimal mix ratio and curing temperature using response surface methodology [29]. Wu investigated the bond strength and pullout energy, curing time, and nano-SiO_2_ content statistical model in ultra-high-strength concrete fiber-matrix bonding performance through response surface methodology [30]. Mohammed studied the response relationship of the tensile properties of high-temperature and nano-silica-modified self-compacting engineering cement composites [31]. Kumar conducted experiments to assess the impact of large volumes of marble sludge on concrete performance using Box-Behnken design, determining the optimal mix through regression analysis and the Derringer’s desirability function method [32]. Zhang utilized Box-Behnken design and simplex centroid design to develop a mixture proportion for permeable concrete with recycled aggregate, validating the model’s effectiveness through verification experiments [33]. Kumar improved the mixture design of concrete by incorporating a large amount of fine waste additives using Box-Behnken design [34]. Guo proposed a nonlinear material parameter inversion model for recycled concrete based on Box-Behnken design, explicitly expressing the implicit relationship between intermediate phase parameters and macroscopic mechanical properties [35]. These studies collectively demonstrate the high analytical precision of the response surface method, highlighting its feasibility in optimizing the mixture proportions of cement-based materials.

The current study explores the use of fly ash and ceramic waste powder as supplementary cementitious materials to replace cement, focusing on evaluating the resistance of the substrate to sulfate attack. The study also aims to quantify the impact of supplementary cementitious materials. Three influencing factors, namely the replacement rate of supplementary cementitious materials, the dosage of ceramic waste powder, and the water-cement ratio, were investigated using a Box-Behnken design experiment within the response surface methodology to analyze the performance of cement-based specimens. The substrate properties were evaluated after curing periods of 7, 28, and 56 days. The mass change rate after 24 cycles of wet-dry alternation in a 5% sodium sulfate solution was established as the model response value to construct the response surface model. Through model analysis of the factors affecting the response value, the mixture proportions of cement-based materials with complex additions of supplementary cementitious materials were optimized. Microscopic testing via SEM was conducted to quantify morphological indicators, providing a quantitative revelation of the mechanism by which complex additions of supplementary cementitious materials enhance the resistance of cement-based materials to sulfate corrosion at the microscopic level. This research outcome effectively utilizes industrial solid waste, reducing natural resource consumption and lowering environmental impact, thus holding significant theoretical implications for the construction industry and industrial sustainability.

## 2. Test materials and processes

### 2.1 Raw materials

The cement used in this study has a strength grade of 42.5, an apparent density of 3160 kg/m³, and a standard consistency water requirement of 26.4%. The SCMs employed include fly ash (FA) and ceramic waste powder (CWP). The fly ash is a Grade II fly ash produced by power plants. The CWP is obtained from the polishing powder of white ceramic tiles produced by a ceramic factory. The X-ray spectroscopy analysis results of the cementitious materials are shown in Table 1.

**Table 1:** X-ray spectroscopy analysis of cementitious materials (mass fraction %).

Element	CaO	SiO_2_	Al_2_O_3_	Fe_2_O_3_	MgO	SO_3_	LOSS
cement	63.10	22.19	5.48	4.23	2.41	2.57	0.03
FA	6.79	47.12	37.92	3.97	0.93	0.69	0.99
CWP	2.7	54.8	32.28	3.76	4.97	0.43	1.06

Table 1 reveals that the CaO content in fly ash is approximately 10.76% of the one in cement. The CaO content in CWP is significantly lower than that in cement, accounting for 4.28% of that in cement. CaO is a crucial component that influences the reactivity of volcanic ash, and compared to cement, the SCMs exhibit lower reactivity [36]. The chemical composition of the SCMs mainly consists of SiO_2_, Al_2_O_3_, and Fe_2_O_3_, with the combined content of these three components in fly ash being 89.01% and in CWP being 90.84%. The SiO_2_ content in fly ash and CWP is 2.12 and 2.47 times that in cement, respectively. The Al_2_O_3_ content is approximately 6.92 and 5.89 times that in cement. Both SiO_2_ and Al_2_O_3_ play a significant role in the formation of C-H-S gel during cement hydration. The SO_3_ content in the SCMs is much lower than that in cement. The selected proportions of the two SCMs in this study are relatively similar, belonging to silica-alumina materials with SiO_2_ and Al_2_O_3_ as the main components. SiO_2_ and Al_2_O_3_ serve as the sources of reactivity in the supplementary cementitious materials.

### 2.2 Raw materials

#### 2.2.1 Material properties

**Fig 1:**
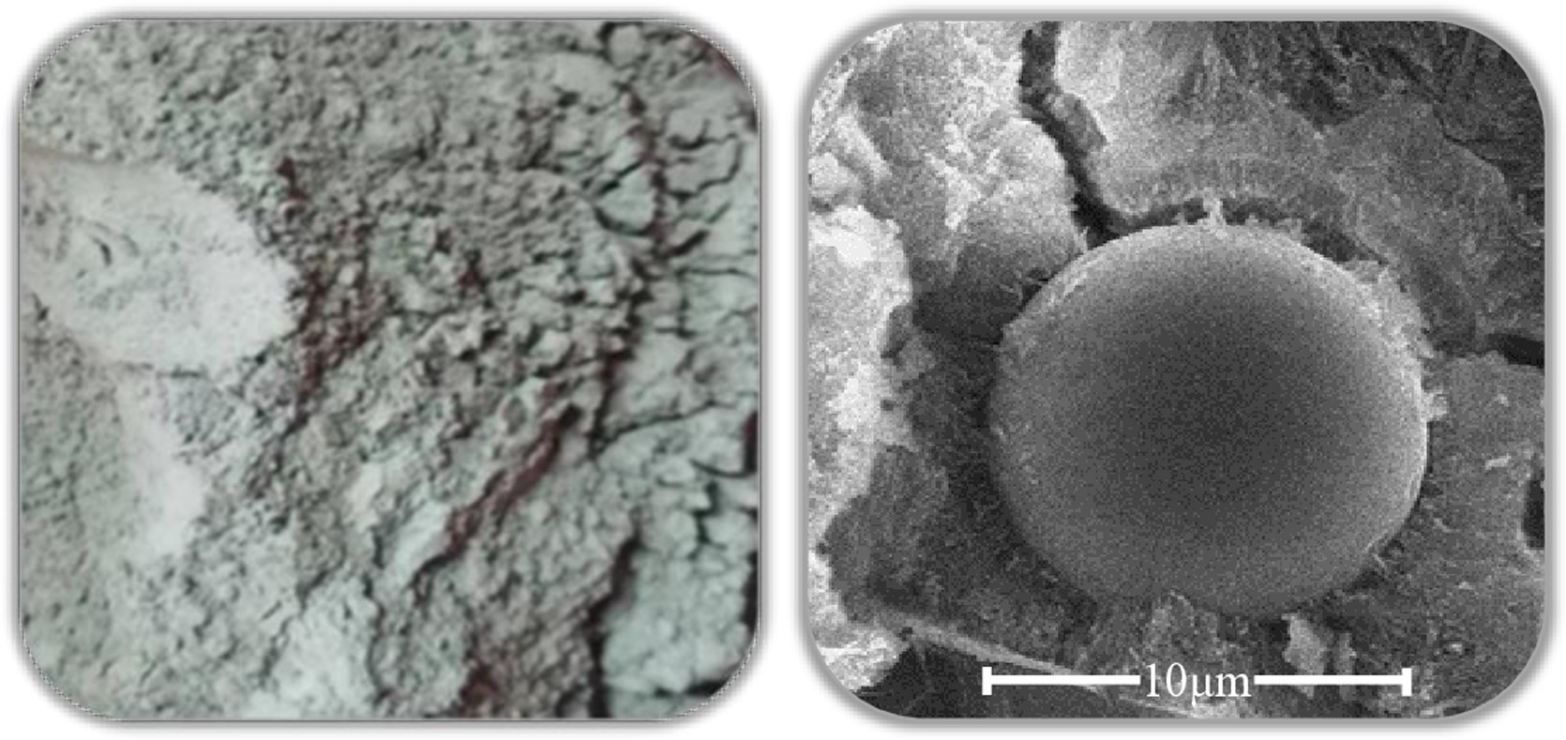
Morphology of fly ash.

**Fig 2:**
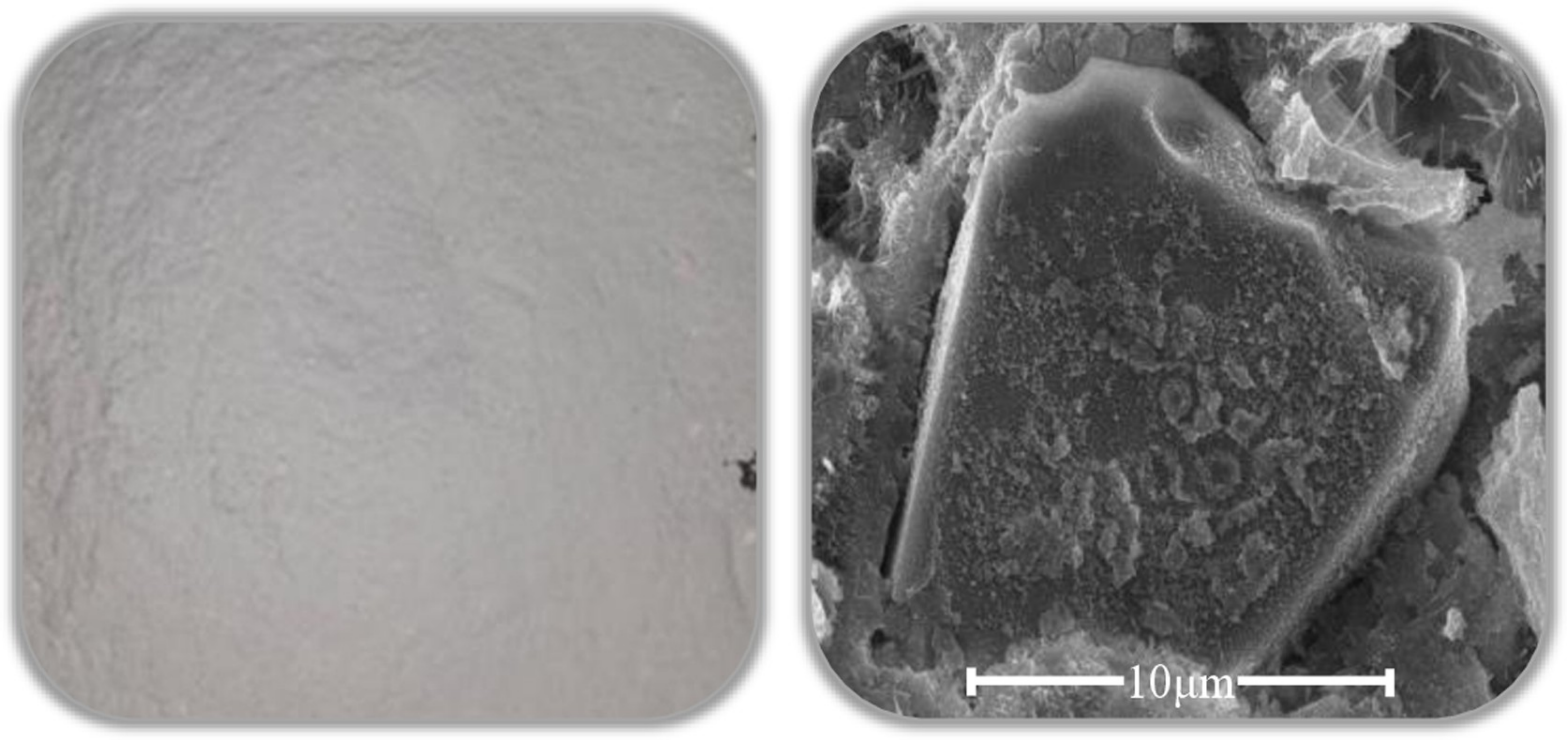
Morphology of ceramic waste powder.

This study conducted SEM testing using the Quanta 250 field emission scanning electron microscope. The equipment, manufactured by the American company FEI, allows for the observation of the morphological structure of polymer materials. Fig 1 displays the morphology of fly ash, with particles exhibiting a spherical shape. Fig 2 illustrates the morphology of ceramic waste powder, presenting an irregular block-like shape. The ceramic waste powder can fill the voids between cement and fine aggregates, and its irregular shape is advantageous for enhancing the compactness of mortar, thereby improving the strength and durability of the mortar.

#### 2.2.2 Particle size analysis

**Fig 3:**
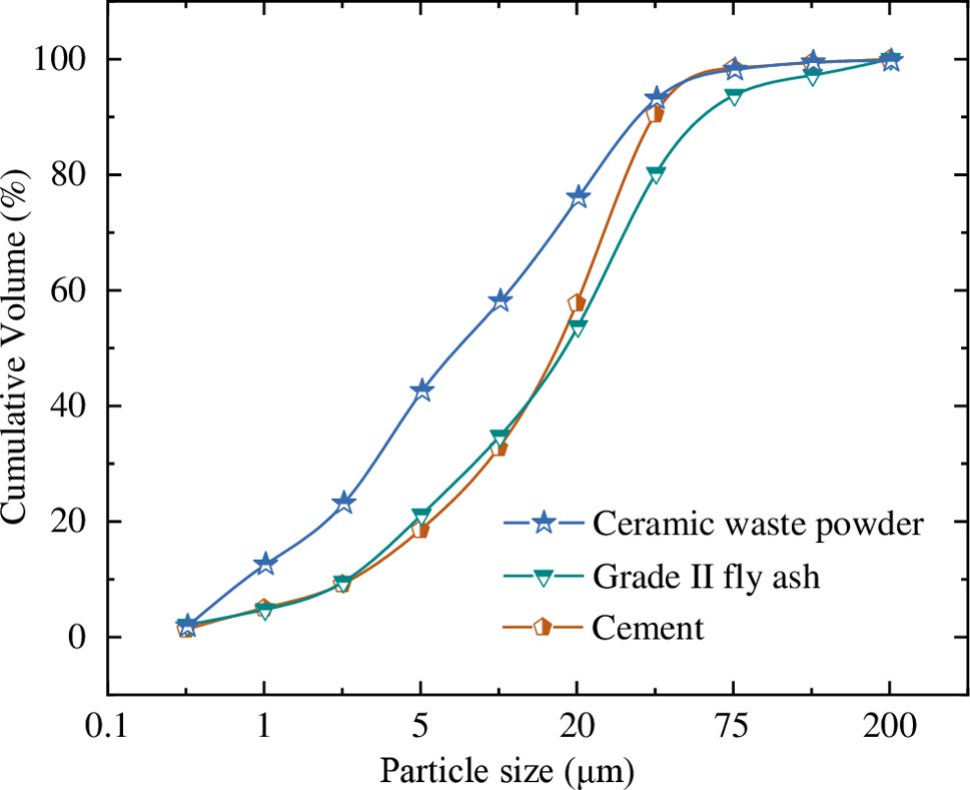
Particle size distribution of cementitious materials.

The particle size distribution was determined using a Mastersizer 2000 laser particle size analyzer, manufactured by Malvern Instruments Ltd. in the United Kingdom. The particle size distribution is shown in Fig 3.

Through laser particle size analysis, it was determined that the particle size distribution of cement and Class II fly ash are similar, with the highest proportion of particles falling in the range of 20–45 μm. The ceramic waste powder exhibits the smallest average particle size, with a median diameter (D50) of 7.29 μm and a specific surface area of 836.2 m^2^/kg. The highest proportion of particles for ceramic waste powder is in the range of 2–5 μm, accounting for 19.41%. The percentage of small particles in ceramic waste powder is lower than in other supplementary cementitious materials. This suggests that ceramic waste powder can directly contribute to the micro-aggregate filling effect on the strength of the cement matrix.

### 2.3 Preparation of specimen

**Fig 4:**
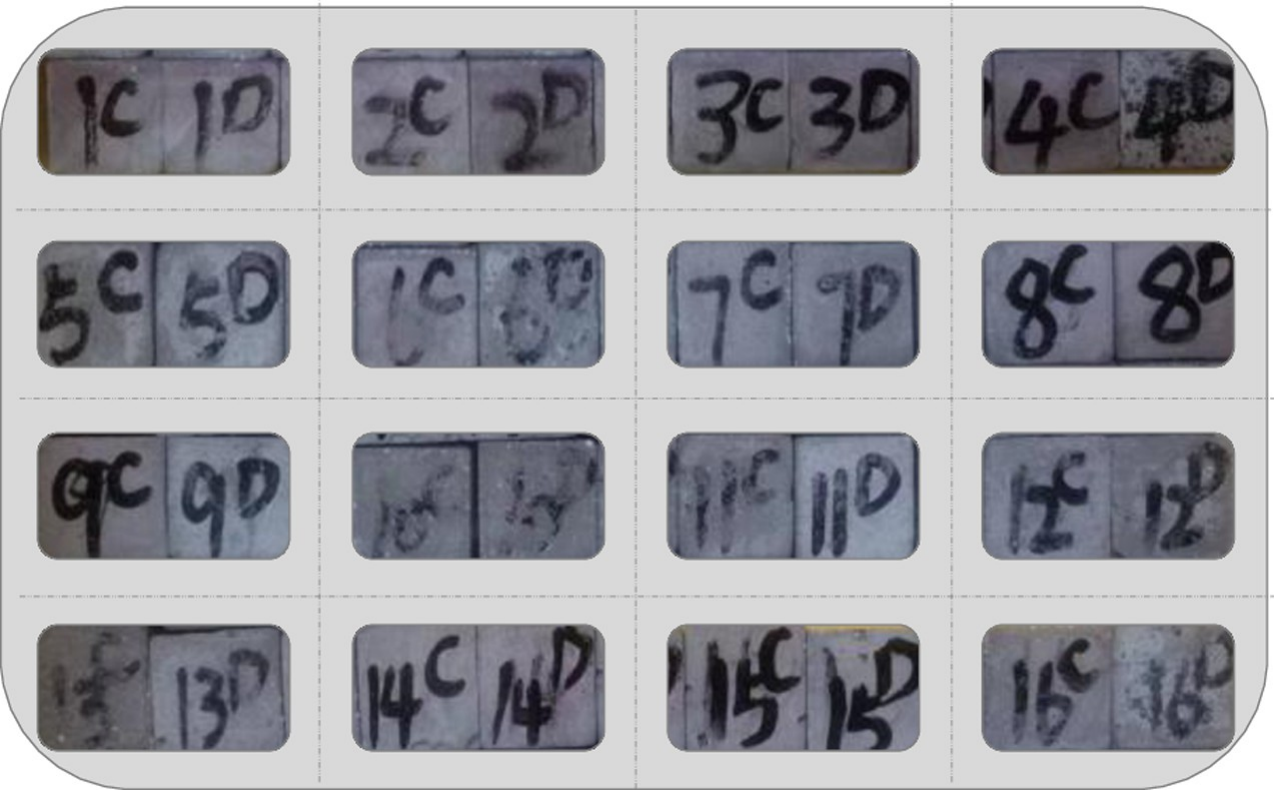
Specimens for the wet-dry cycle -sulfate erosion test.

The cement paste specimens in this study had dimensions of 20 mm × 20 mm × 20 mm, with a total of 12 specimens for each mix proportion. The preparation process followed the " Standard for test method of mechanical properties on ordinary concrete " [37]. Firstly, the required materials were placed in a mixer according to the mix proportion and dry mixed for 30 seconds. Then, a fixed amount of water was added uniformly, and the mixture was further mixed for 2–3 minutes. After mixing, the mixture was poured into molds and compacted using a vibrating table. After 24 hours of curing at room temperature, the specimens were de-molded and placed in a standard curing room. The curing ages were 7 days, 28 days, and 56 days, respectively. At the end of each curing age, four specimens were taken from each group of mixed proportions. Based on the preliminary test conclusions of the research group, a total of 24 cycles of wet-dry and sulfate erosion tests were conducted. The control group consisted of specimens with the same mix proportion that were immersed in water. Fig 4 shows the test specimen.

### 2.4 Experimental design

The experimental design was conducted using the Box-Behnken design in the response surface model. Three factors were selected for the design, which included the proportion of SCMs (FA and CWP) replacing an equal mass of cement (Factor A), the ratio of CWP to the mass of SCMs (Factor B), and the water-to-binder ratio (Factor C). The mass change rates of the materials under the conditions of 24 cycles of wet-dry and sulfate erosion with curing ages of 7 days, 28 days, and 56 days were taken as the three sets of response values for the model.

Based on the existing research and considering factors such as the feasibility of the trial mix, the specific range of Factor A was determined. Previous studies have investigated the properties of SCMs replacing cement. The range studied varied from 0% to 70%. The results indicated that the optimal mechanical performance was achieved with a substitution rate of 10% to 20% [21], while the best resistance to sulfate erosion was observed at a substitution rate of 30% [27]. Therefore, the range for Factor A was determined to be within the interval of 10% to 30% based on these findings.

Factor B investigates the interaction effect of dual-blended SCMs. A summary of the literature is presented in Table 2. Table 2 provides a compilation of suggested percentages for different dosages of CWP.

**Table 2:** Research conclusions of CWP dosage.

References	Amount of ceramic waste powder	Recommended value
A Heidari [38]	0, 10%, 15%, 20%, 25%, 30%, 40%	Decreasing the amount of admixture
S Subaşı [39]	0, 5%, 10%, 15%, 20%	15%
XY Chen [40]	0, 10%, 20%, 30%, 40%	10% - 20%
L Li [41]	0, 10%, 20%	10%

Heidari et al. found that the corrosion intensity loss of CWP-based materials ranged from 10% to 20% under long-term curing conditions [38]. Subaşı suggested that the comprehensive performance of cement-based materials is improved when the substitution rate of CWP reaches 15% [39]. Chen’s research demonstrated that replacing 10% to 20% of cement with CWP can enhance the long-term strength (56 days) of recycled concrete [40]. Li et al. confirmed that the compressive strength increased by 8.75% when 10% of cement was replaced with CWP, and the compressive strength variation remained within an acceptable range when the substitution rate was 20% [41]. Taking into account the findings of the comprehensive literature review, CWP can be used as an SCM to replace cement by no more than 20% in mass. Considering the range of Factor A, the range for the substitution rate of CWP to the mass of SCM is determined to be 25% to 75%.

In cementitious mixtures, the water-to-binder ratio is a critical factor affecting the compactness of the matrix. Insufficient water content leads to incomplete hydration of cement and affects the strength. Excessive water content results in the presence of free water in the hardened cement matrix, which evaporates and forms voids that are detrimental to long-term durability. The specifications require a water-to-binder ratio of less than 0.55 for cementitious materials with waterproofing properties. Based on the previous achievements of the research group, the experimental level for Factor C was determined to be within the range of 0.38 to 0.53. The levels of the experimental factors are shown in Table 3. A total of 17 groups of experiments were conducted, and the design of the experimental mix proportions is presented in Table 4.

**Table 3:** Test factors and levels.

Level	Design factor (%)		
	Cement	B	C
-1	10	25	0.38
0	20	50	0.455
1	30	75	0.53

**Table 4:** Test design.

Number	Design factor						
	A (%)	B (%)	C (%)	Cement(kg/m^3^)	Fly ash(kg/m^3^)	CWP(kg/m^3^)	Water (kg/m^3^)
1	20	50	0.455	638.4	79.8	79.8	665
2	30	50	0.38	634.9	136.05	136.05	556
3	20	50	0.455	638.4	79.8	79.8	665
4	20	50	0.455	638.4	79.8	79.8	665
5	30	25	0.455	558.6	59.85	179.55	665
6	20	75	0.53	550.4	103.2	34.4	775
7	30	75	0.455	558.6	179.55	59.85	665
8	10	75	0.455	718.2	59.85	19.95	665
9	10	50	0.38	816.3	45.35	45.35	556
10	10	50	0.53	619.2	34.4	34.4	775
11	10	25	0.455	718.2	19.95	59.85	665
12	20	50	0.455	638.4	79.8	79.8	665
13	20	25	0.53	550.4	34.4	103.2	775
14	20	25	0.38	725.6	45.35	136.05	556
15	20	75	0.38	725.6	136.05	45.35	556
16	20	50	0.455	638.4	79.8	79.8	665
17	30	50	0.53	481.6	103.2	103.2	775

### 2.5 Experimental and testing methods

#### 2.5.1 Dry-wet cycle-sulfate erosion test method

The specimens were cured until the designated age and then dried in air for 24 hours, with the resulting mass recorded as M0. A 5% Na_2_SO_4_ solution was prepared, ensuring a pH value between 6 and 8. Subsequently, the specimens were fully immersed in the sodium sulfate solution for 24 hours followed by natural air drying for another 24 hours, constituting one cycle of wet-dry alternation, with the mass denoted as M_n_.

#### 2.5.2 Corrosion resistance coefficient

According to the "Test method for determining the capability of resisting sulfate corrode of cement " [42], the corrosion resistance coefficient of cementitious materials is defined, as shown in Eq (1). The corrosion resistance coefficient is equal to the compressive strength test value *k*_*c*_ (MPa) of the cementitious material immersed in the corrosive solution divided by the corresponding compressive strength test value *k*_*c*_ (MPa) of the cementitious material cured in water for the corresponding age.


$$\gamma =k_{c}/k_{w}$$ (1)

#### 2.5.3 Mass loss

After completing each wet-dry cycle of sulfate erosion, the mass of the specimens was measured using an electronic balance with a precision of 0.1g. The mass loss rate was calculated according to Eq (2).


$$M_{\text{r}}=\frac{M_{\text{n}}-M_{0}}{M_{0}}$$ (2)

In the equation, *M*_r_ represents the average mass loss of the specimens (%), *M*_*n*_ represents the average mass after *n* cycles of wet-dry exposure, and *M*_0_ represents the average initial mass of the specimens. The mass loss rate can serve as an indicator of whether the cementitious specimens are damaged. According to the "Standard for test methods of long-term performance and durability of ordinary concrete" [43], when the mass loss rate exceeds 5%, the specimens are considered to be damaged or fail to meet the requirements of practical engineering.

#### 2.5.4 SEM analysis

The SEM testing was conducted using the Quanta 250 scanning electron microscope manufactured by FEI Company in the United States. The specimens were crushed into smooth and angular fragments. They were immersed in anhydrous ethanol to halt hydration. Before testing, the crushed fragments were taken out and ensured to have a smooth and clean surface. They were then placed in an oven and dried at 40°C for 10 hours. Before testing, the specimens were subjected to gold spraying treatment.

## 3. Results and analysis

### 3.1 Corrosion morphology analysis

**Fig 5:**
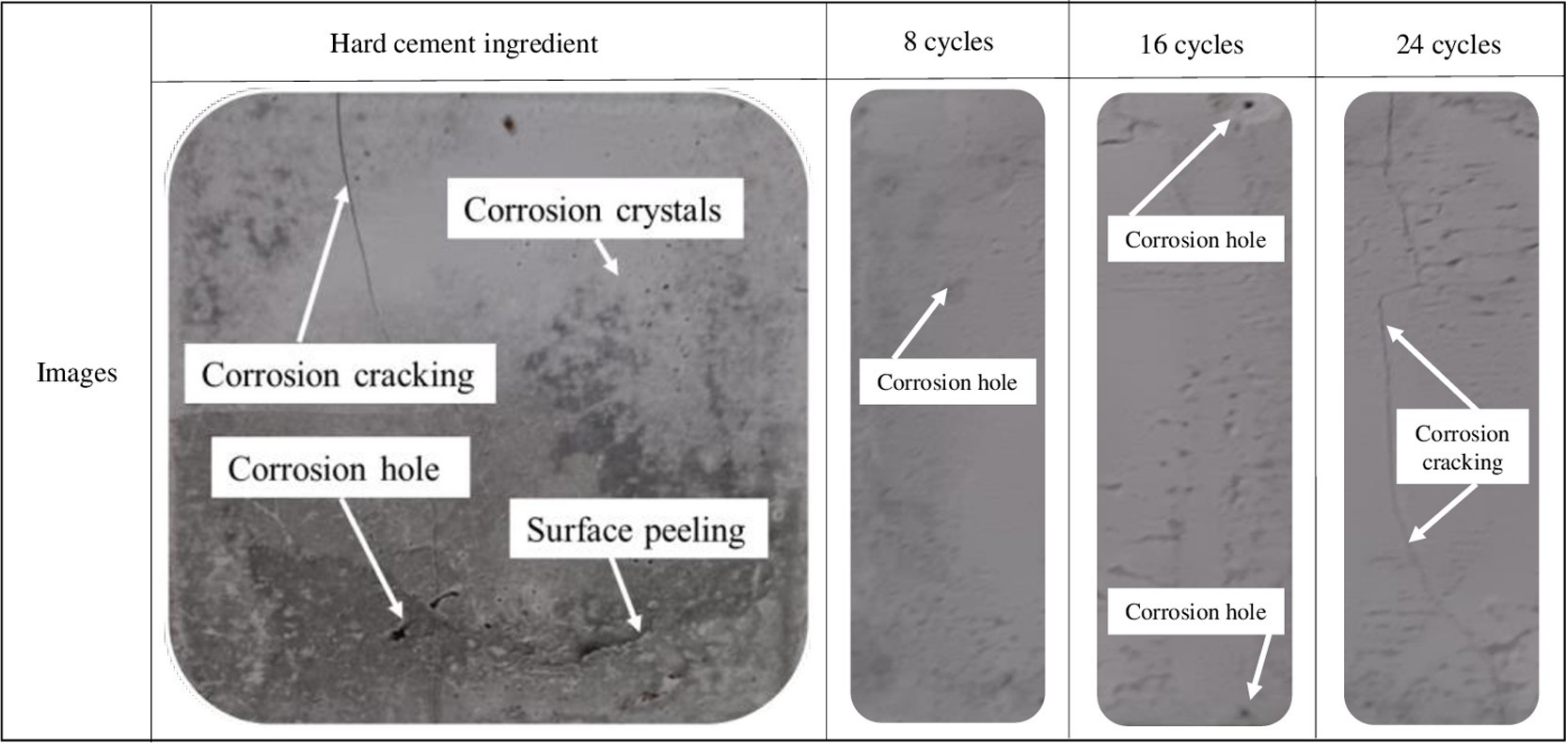
Corrosion morphology of cement slurry.

Fig 5 presents the typical morphological image of cement paste specimens from the control group after 28 days of curing and corrosion.

From Fig 5, it can be observed that the surface of the cementitious material becomes rough after corrosion, with localized peeling and the presence of white deposits as corrosion crystals. The corrosion cracks lengthen and widen as the corrosion time increases. After corrosion, there are visible pores. The changes in the typical morphological images after different cycles of wet-dry exposure are as follows: After 8 cycles, the surface becomes relatively smooth, with uneven distribution of corrosion crystals. The crystal layer is thin, allowing the original gray color of the cementitious material to show through. There are no significant corrosion cracks, but the corrosion causes unevenness on the surface without deep penetration. After 16 cycles, the corrosion deepens, resulting in a rough surface. The pits formed by corrosion become more densely distributed, and fine cracks appear but do not penetrate the entire section. After 24 cycles, the corrosion area expands and almost covers the entire surface layer. Localized corrosion pits deepen, forming holes, and prominent penetrating cracks become evident.

### 3.2 Analysis of corrosion resistance coefficient

**Fig 6:**
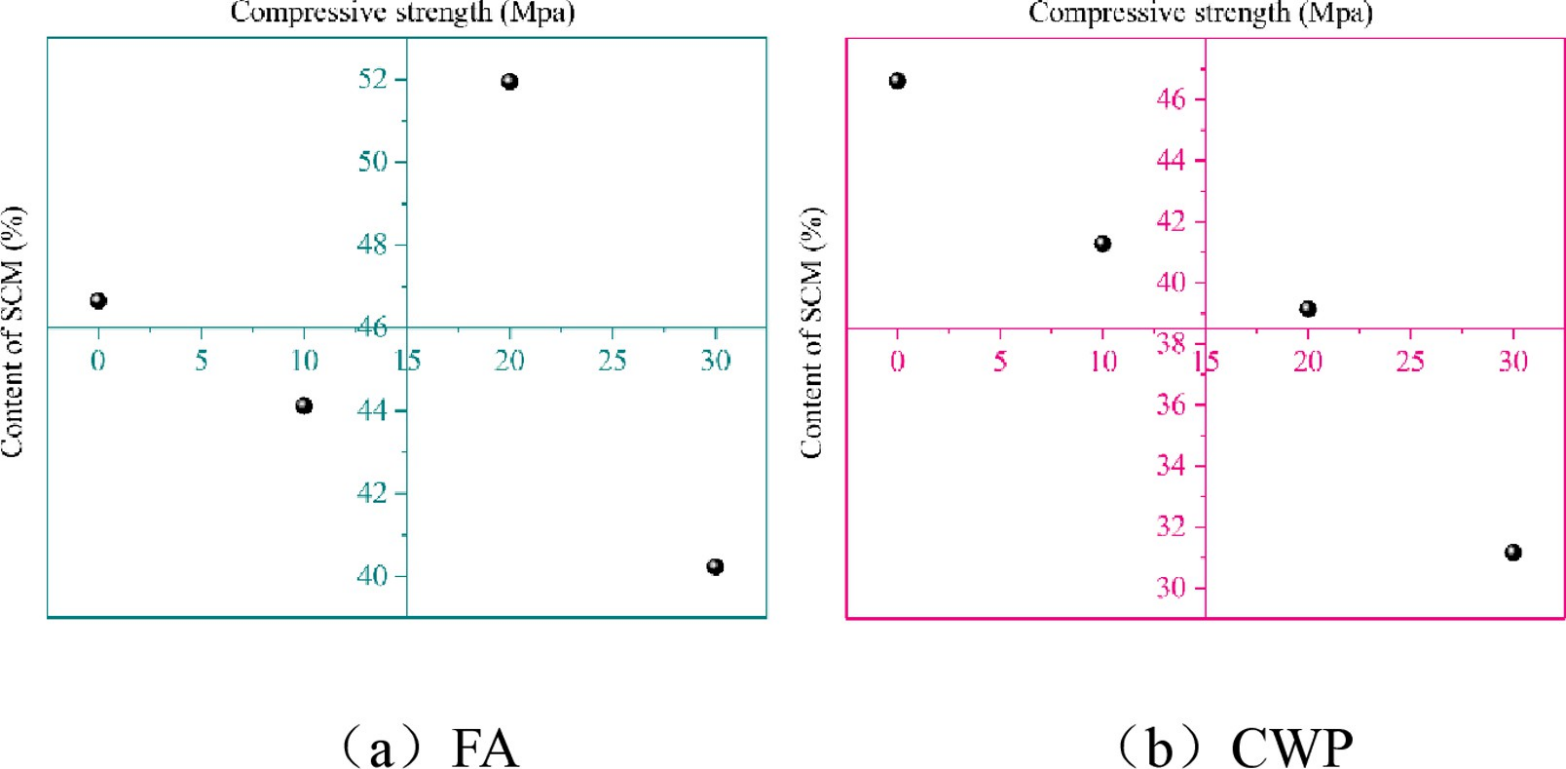
Relationship between SCMs and compressive strength (water immersion); (a)FA; (b)CWP.

Fig 6 displays the relationship between the dosage of SCMs and the compressive strength of specimens cured for 28 days and immersed in water for 48 days.

According to Fig 6(A), it can be observed that the compressive strength is highest when the dosage of fly ash is 20%, with a strength increase of 11.34% compared to the reference concrete. The compressive strength of specimens with FA dosages of 10% and 30% is lower than that of the reference concrete. This demonstrates that an appropriate dosage of FA can improve the strength of cementitious materials, possibly due to the reduction in the content of C_3_A and C_3_S after FA substitution, which in turn enhances the strength [44]. From Fig 6(B), it can be seen that with the increase in CWP dosage, the immersed compressive strength significantly decreases, consistent with the findings of reference [45]. The rate of decline is slowest between 10% and 20% substitution levels. The activity of CWP is lower than that of cement, and as the dosage of SCM increases, the binder system is unable to generate sufficient hydration products to fill the voids, resulting in insufficient density of the cementitious matrix and a decrease in strength.

**Fig 7:**
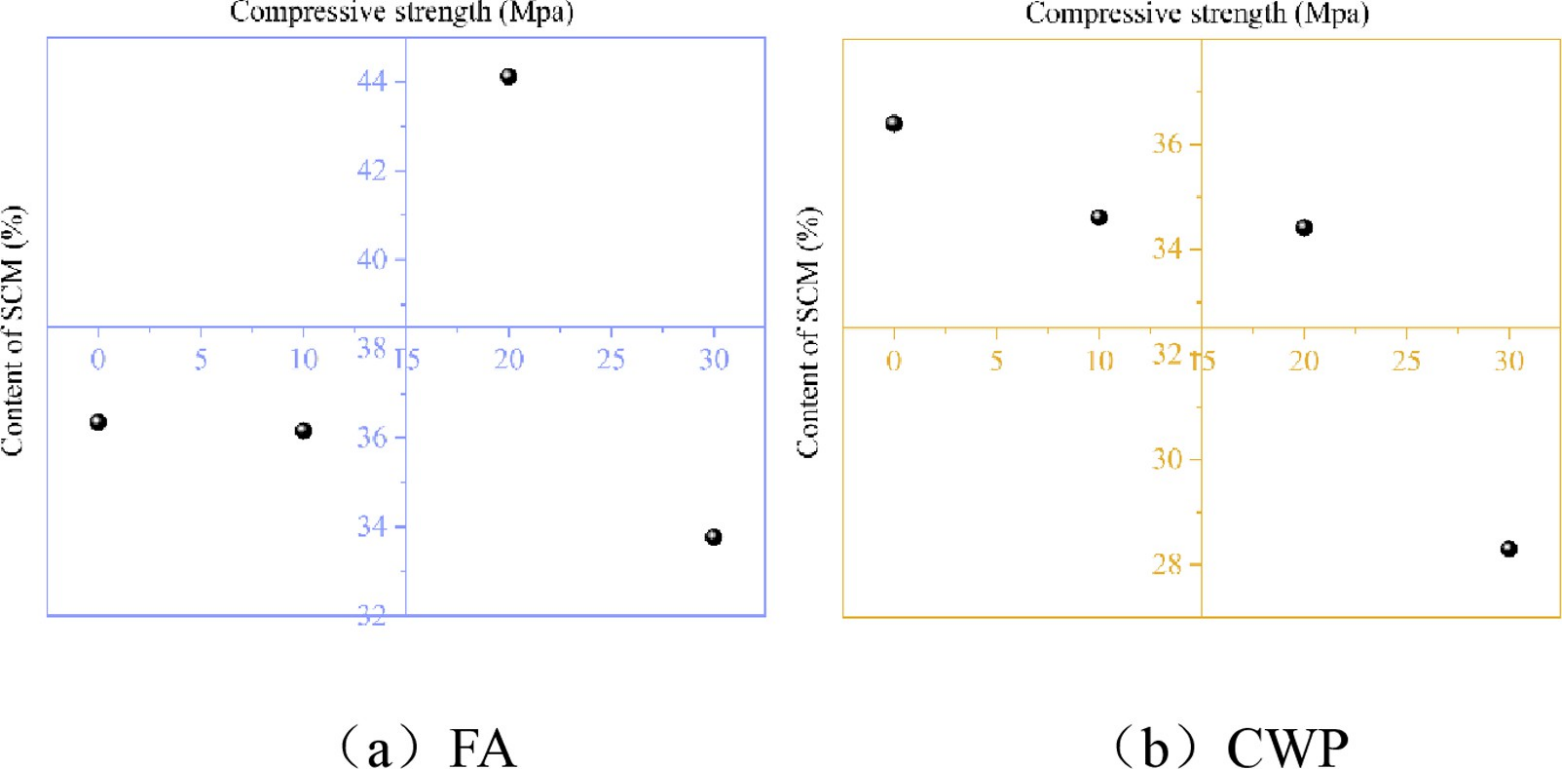
Relationship between SCMs and compressive strength (corrosion condition); (a)FA; (b)CWP.

Fig 7 illustrates the relationship between the dosage of SCMs and the compressive strength of specimens cured for 28 days and subjected to 24 cycles of wet-dry exposure and sulfate erosion.

**Fig 8:**
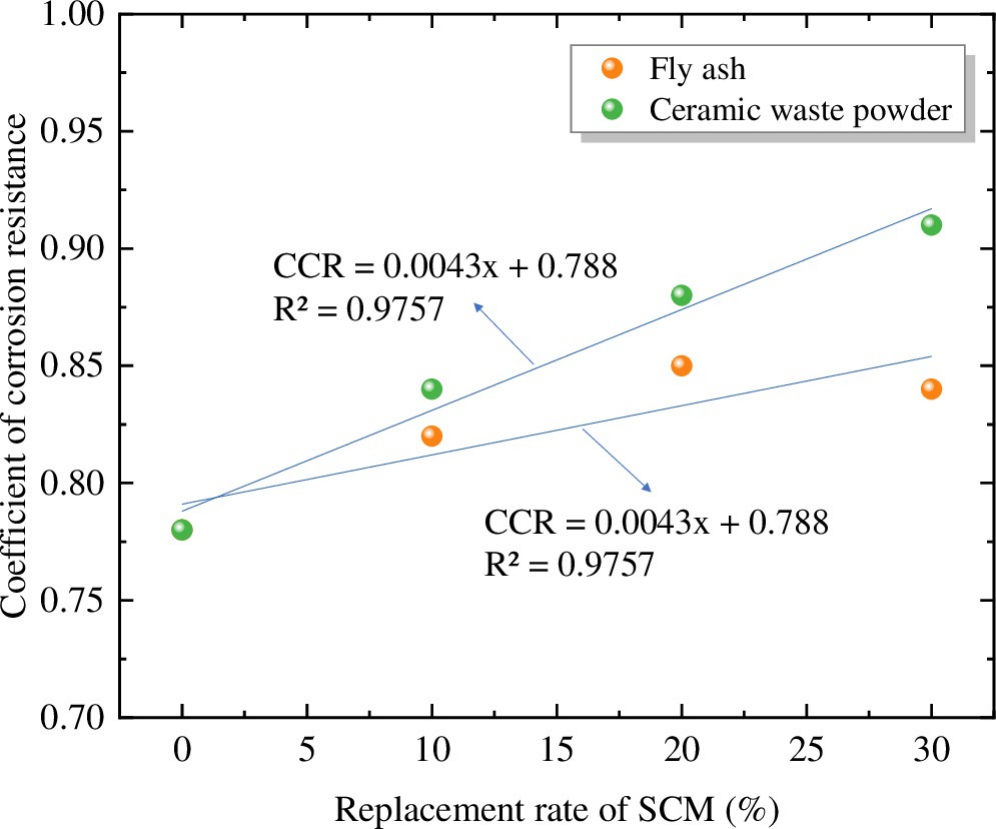
Relationship between corrosion resistance coefficient and SCM content.

Fig 7(A) presents the compressive strength of specimens subjected to 24 cycles of wet-dry exposure and sulfate erosion, with FA substitution ranging from 0% to 30%. From Fig 7(A), it can be observed that the strength of the same mixture ratio of the base material decreases in the wet-dry and corrosion environment compared to immersion in water. As the mass of FA substitution increases, the degree of strength reduction decreases, indicating that FA can enhance the corrosion resistance of the matrix. Fig 7(B) displays the compressive strength of specimens subjected to 24 cycles of wet-dry exposure and sulfate erosion, with CWP substitution ranging from 0% to 30%. It can be seen from the figure that the strength decreases in the wet-dry and corrosion environment compared to immersion in water, and the degree of reduction diminishes as the mass of CWP substitution increases. Compared to FA, ceramic micro-powder exhibits higher resistance to wet-dry exposure and sulfate erosion when substituted for an equal mass of cement [46]. Fig 8 depicts the relationship between the erosion resistance coefficient and the dosage of SCMs replacing cement.

According to Fig 8, it can be observed that as the mass of FA substitution increases, the corrosion resistance significantly improves. However, after exceeding a substitution level of 20%, the corrosion resistance slightly decreases. On the other hand, with an increase in the mass of CWP substitution, the corrosion resistance linearly improves. Overall, the cementitious matrix with CWP exhibits higher corrosion resistance compared to FA. Based on the particle size analysis results in Section 2.2.2, CWP differs significantly from cement particles, resulting in better micro-filling and morphological effects, thereby further enhancing the density of the matrix. This conclusion aligns with the findings of reference [47].

### 3.3 Response surface analysis of mass loss

The mass variation rate of the cementitious matrix after 24 cycles of wet-dry exposure and sulfate erosion, with curing ages of 7 days, 28 days, and 56 days, is taken as the response variable in the model. A total of 17 groups were tested. The experimental design and results are shown in Table 5.

**Table 5:** Response surface design and results.

Number	Design factor (%)			Rate of mass change (%)		
	A	B	C	F-7	F-28	F-56
1	20	50	0.455	5.67	1.35	0.00
2	30	50	0.380	2.92	0.00	-2.07
3	20	50	0.455	5.11	1.41	-0.71
4	20	50	0.455	5.97	1.4	0.00
5	30	25	0.455	6.50	-0.73	-1.48
6	20	75	0.530	12.61	0.00	-2.24
7	30	75	0.455	7.87	0.00	-1.52
8	10	75	0.455	6.67	-0.71	0.00
9	10	50	0.380	3.42	0.66	-0.65
10	10	50	0.530	13.04	1.56	-0.78
11	10	25	0.455	7.58	1.42	0.00
12	20	50	0.455	5.74	0.74	-0.75
13	20	25	0.530	7.56	2.42	-1.53
14	20	25	0.380	4.35	0.69	-0.68
15	20	75	0.380	2.00	0.66	-1.33
16	20	50	0.455	4.62	1.46	-0.72
17	30	50	0.530	9.57	1.6	-2.33

#### 3.3.1 Response surface model

The experimental results were fitted using the Box-Behnken model. The quadratic polynomial regression model between the three factors and the response variable is shown in Eq (3):

$$Y=\beta_{0}+\sum_{i=1}^{n}\left(\beta_{i}x_{i}\right)+\sum_{i=1}^{n}\left(\beta_{ii}{x_{\text{i}}}^{2}\right)+\sum_{i=1}^{n}\sum_{j=1}^{n}\left(\beta_{ij}x_{\text{i}}x_{j}\right)+\varepsilon$$ (3)

In the equation, *Y* represents the predicted value of the response variable, *x*_*i*_ represents the coded value of the selected variables, ${x_{i}}^{2}$ represents the quadratic effects of the factors, *x*_*i*_*x*_*j*_ represents the interaction effects between factors, *β*_0_ represents the constant regression coefficient, *β*_*i*_ represents the regression coefficient of the linear effects term, *β*_*ii*_ represents the regression coefficient of the quadratic effects term, *β*_*ij*_ represents the regression coefficient of the interaction effects term, *ε* represents the random error, and *n* represents the number of variables.

The quality loss of the cementitious matrix is determined by the proportions of SCMs, the mass ratio of CWP to the SCMs, and the water-to-binder ratio. The experimental data in Table 5 were subjected to regression analysis. A quadratic polynomial regression equation was established to model the mass variation of the cementitious matrix at 7 days, 28 days, and 56 days under wet-dry exposure and sulfate erosion conditions, as shown in Table 6.

**Table 6:** Prediction model of mass loss rate.

Curing period (d)	Prediction model of mass loss rate
7	Y_7_ = 29.17–0.18A+-0.57B+-83.77C+0.0023AB-0.99AC+0.99BC
	+0.012A²+0.00**1**B²+114.71C²
28	Y_28_ = 4.27–0.02A+0.17B-33.78C+0.003AB+0.23AC-0.32BC
	-0.006A²-0.001B²+56.04C²
56	Y_56_ = -30.42+0.012A+0.022B+136.53C-0.00004AB-0.04AC-0.008BC-0.002A²-0.0002B²-152.58C²

#### 3.3.2 Model significance analysis

The significance of the established regression model was tested through analysis of variance (ANOVA), and the significance of the linear effects, quadratic effects, and interaction effects of each factor on the response variable was analyzed. The results of the ANOVA for the regression model are shown in Tables 8–10. In the ANOVA for the regression model, F represents the variance of the error caused by the variation of the corresponding factor. The significance of the model is determined by both the F value and the p value. A smaller p-value (≤0.05) and a larger F value indicate a more significant model [48].

According to Table 7, the F value of the model is 23.84 with a P value of 0.0002, indicating that the model is significant, and the effects of the factors on the corrosion resistance of the 7-day cured specimens are significant. The P value for the individual factor C is less than 0.0001, indicating that the water-to-binder ratio has an extremely significant effect on the mass variation rate of the cementitious matrix under wet-dry exposure and sulfate erosion conditions. The P value for the interaction effect BC is 0.0027, indicating a significant interaction and coordination between the substitution ratio of CWP for SCMs and the water-to-binder ratio. The P value for the quadratic term A^2^ is 0.0217, indicating a significant impact on the results. Reducing the water-to-binder ratio not only refines the pore size distribution towards smaller pores in the cement paste but also significantly reduces the total pore volume, especially the harmful large pores, thereby significantly improving the resistance of the cementitious matrix to sulfate erosion [49].

**Table 7:** Analysis of variance of the regression model (F-7).

Category	Some of squares	Degree of freedom	Mean square	F value	P value	Significance
Model	143.21	9	15.91	23.84	0.0002	significant
A	1.85	1	1.85	2.78	0.1396	
B	1.25	1	1.25	1.87	0.2137	
C	113.18	1	113.18	169.57	< 0.0001	
AB	1.3	1	1.3	1.95	0.2056	
AC	2.21	1	2.21	3.3	0.1119	
BC	13.69	1	13.69	20.51	0.0027	
A²	5.77	1	5.77	8.64	0.0217	
B²	1.33	1	1.33	2	0.2004	
C²	1.75	1	1.75	2.63	0.1491	
Lack of Fit	3.47	3	1.16	3.84	0.1132	not significant

Based on the conclusions drawn from Table 8, the F value of the model is 10.63 with a P value of 0.0025, indicating that the model is significant, and the effects of the factors on the corrosion resistance of the 28-day cured specimens are significant. The individual factors B and C have P values less than 0.05, with B having a greater impact than C. This suggests that the addition of SCMs and the proportion of CWP have a more significant effect on the corrosion resistance of the cementitious specimens at 28 days of curing. Under the interaction effects of the factors, the P value for the two-factor interaction AB is 0.0044, and BC is 0.0106, demonstrating that the interaction effects of AB and BC have a significant impact on the experimental results. The quadratic terms A^2^ and B^2^ also have a significant influence on the corrosion resistance of the matrix. The reactive activity of the SCMs is stimulated under the 28-day curing conditions, promoting their secondary hydration reaction [19], thereby reducing the corrosion-induced mass loss of the cementitious matrix.

**Table 8:** Analysis of variance of the regression model (F-28).

Category	Some of squares	Degree of freedom	Mean square	F value	P value	Significance
Model	11.45	9	1.27	10.63	0.0025	significant
A	0.5305	1	0.5305	4.43	0.0732	
B	1.85	1	1.85	15.49	0.0056	
C	1.59	1	1.59	13.32	0.0082	
AB	2.04	1	2.04	17.09	0.0044	
AC	0.1225	1	0.1225	1.02	0.3453	
BC	1.43	1	1.43	11.94	0.0106	
A²	1.68	1	1.68	14.07	0.0072	
B²	1.75	1	1.75	14.63	0.0065	
C²	0.4185	1	0.4185	3.5	0.1036	
Lack of Fit	0.4775	3	0.1592	1.77	0.2918	not significant

According to Table 9, the F value of the model is 6.26 with a p-value of 0.0122, indicating that the model is significant, and the effects of the factors on the corrosion resistance of the 56-day cured specimens are significant. The individual factor A and the quadratic term C^2^ have P values less than 0.05, indicating a significant impact on the experimental results. The hydration products of cement clinker contain a large amount of hydrated calcium aluminate and calcium hydroxide, which are the main sources of corrosion products such as ettringite and gypsum. Increasing the dosage of SCMs effectively reduces the amount of cement used, resulting in a decrease in the generation of corrosion products and a reduction in the degree of corrosion in the cementitious matrix.

**Table 9:** Analysis of variance of the regression model (F-56).

Category	Some of squares	Degree of freedom	Mean square	F value	P value	Significance
Model	8.75	9	0.9723	6.26	0.0122	significant
A	4.46	1	4.46	28.69	0.0011	
B	0.245	1	0.245	1.58	0.2494	
C	0.5778	1	0.5778	3.72	0.0951	
AB	0.0004	1	0.0004	0.0026	0.9609	
AC	0.0042	1	0.0042	0.0272	0.8736	
BC	0.0009	1	0.0009	0.0058	0.9414	
A²	0.1122	1	0.1122	0.7226	0.4234	
B²	0.0957	1	0.0957	0.6162	0.4582	
C²	3.1	1	3.1	19.97	0.0029	
Lack of Fit	0.4525	3	0.1508	0.9509	0.4962	not significant

The Lack of Fit F values for the combined curing periods of 3 days, 28 days, and 56 days are all greater than 0.05, indicating that the lack of fit terms is not significant, and the experimental results have a high level of credibility with no significant differences caused by the experimental factors.

The reliability analysis results of the model are shown in Table 10. The coefficient of determination (R²) represents the degree of difference between the predicted values and the true values of the regression model response. The value ranges from 0 to 1, and a value closer to 1 indicates a better fit of the regression model. The Adjusted R², which also ranges from 0 to 1, represents the goodness of fit of the model, with a higher value indicating a better fit. The Adeq Precision reflects the model’s ability to resist interference, and a value greater than 4 indicates that the model is reasonable.

**Table 10:** Model reliability analysis table.

Model	R²	Adjusted R²	Adeq Precision
Y_7_	0.9684	0.9278	17.9107
Y_28_	0.9318	0.8442	12.8597
Y_56_	0.8895	0.7474	8.8217

According to Table 10, the R² values for the Y_7_, Y_28_, and Y_56_ models are 0.9684, 0.9318, and 0.8895, indicating a good fit of the regression models. The Adjusted R² values are 0.9278, 0.8442, and 0.7474. The Adeq Precision values are all greater than 4, indicating that the models have high reliability and fitting accuracy.

**Fig 9:**
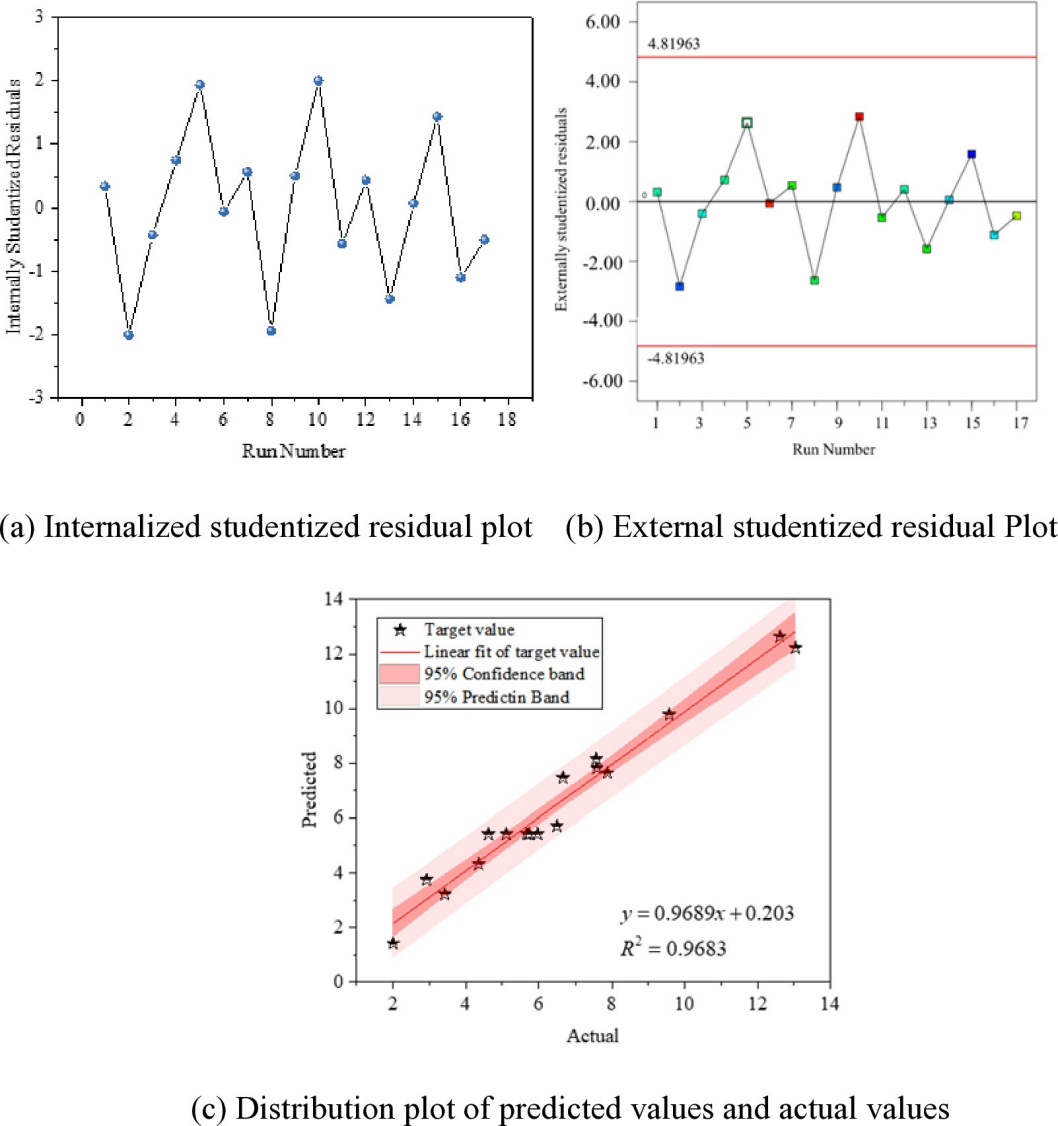
Fitting of mass change rate in 7 days; (a) Internalized studentized residual plot; (b) External studentized residual Plot; (c) Distribution plot of predicted values and actual values.

**Fig 10:**
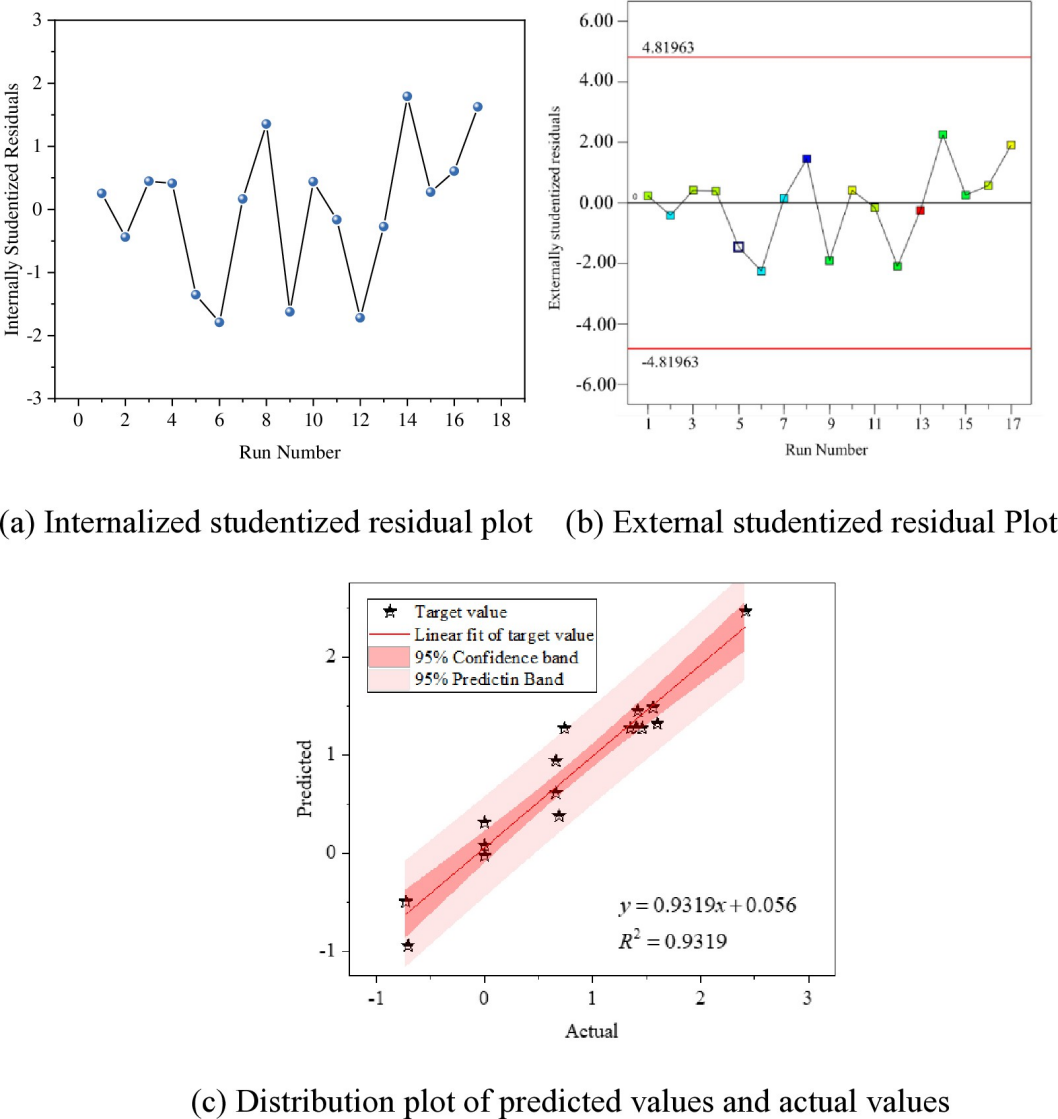
Fitting of mass change rate in 28 days; (a) Internalized studentized residual plot; (b) External studentized residual Plot; (c) Distribution plot of predicted values and actual values.

**Fig 11:**
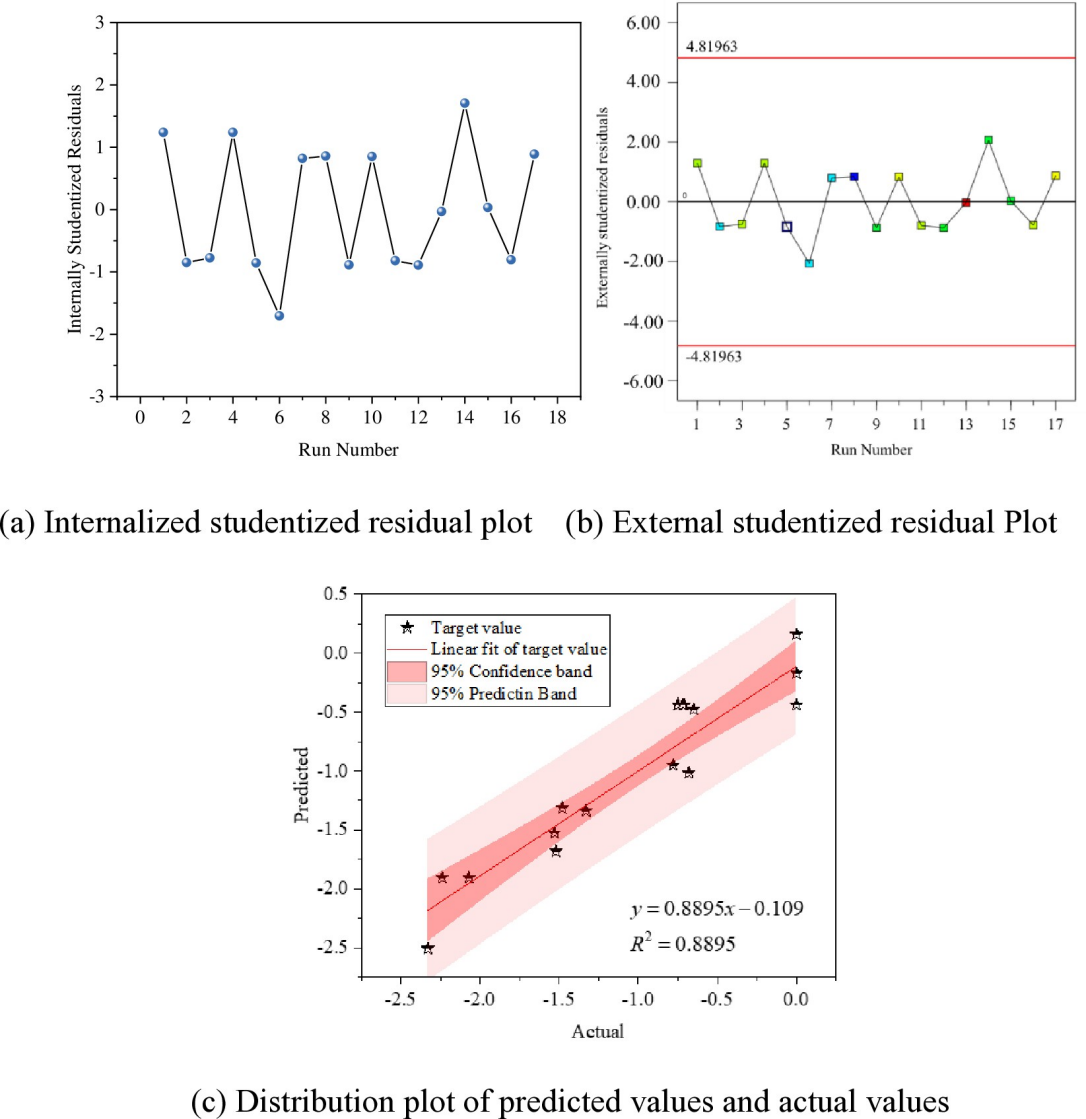
Fitting of mass change rate in 56 days; (a) Internalized studentized residual plot; (b) External studentized residual Plot; (c) Distribution plot of predicted values and actual values.

The fitting results of the models for the mass variation rates at 7 days, 28 days, and 56 days are shown in Figs 9–11.

Figs 9–11 (A) the distribution plot of the experimental model data is presented. Generally, an ISR absolute value higher than 3 is considered an outlier. It is observed from the plot that all data points are accurate without any outliers. To ensure the accuracy of residuals, the Externally Studentized Residual method, which involves excluding the data point itself when calculating the standard error for each data element, is employed. The Externally Studentized Residual conforms to a t-distribution with degrees of freedom of N-k-2. The adherence of the model data’s Externally Studentized Residual to a t-distribution is used to assess the uniqueness of the experimental values.

In Figs 9–11 (B) illustrates that the Externally Studentized Residuals of the model fall within the upper and lower limits of the systematically calculated t-distribution, indicating the accuracy of the residual data points.

Figs 9–11 (C), each data point is closely distributed near the diagonal line, displaying an approximate linear distribution. To further investigate the relationship between predicted and measured values, linear fitting of the data is carried out, with fitting accuracies exceeding 0.88, indicating a significant linear relationship. Additionally, all data points fall within the 95% confidence interval and 95% prediction band, validating the applicability of the fitting results. This confirms the high reliability of the response values predicted using the model, enabling the prediction of actual test results through the established regression model.

#### 3.3.3 Interaction analysis of significant factors

**Fig 12:**
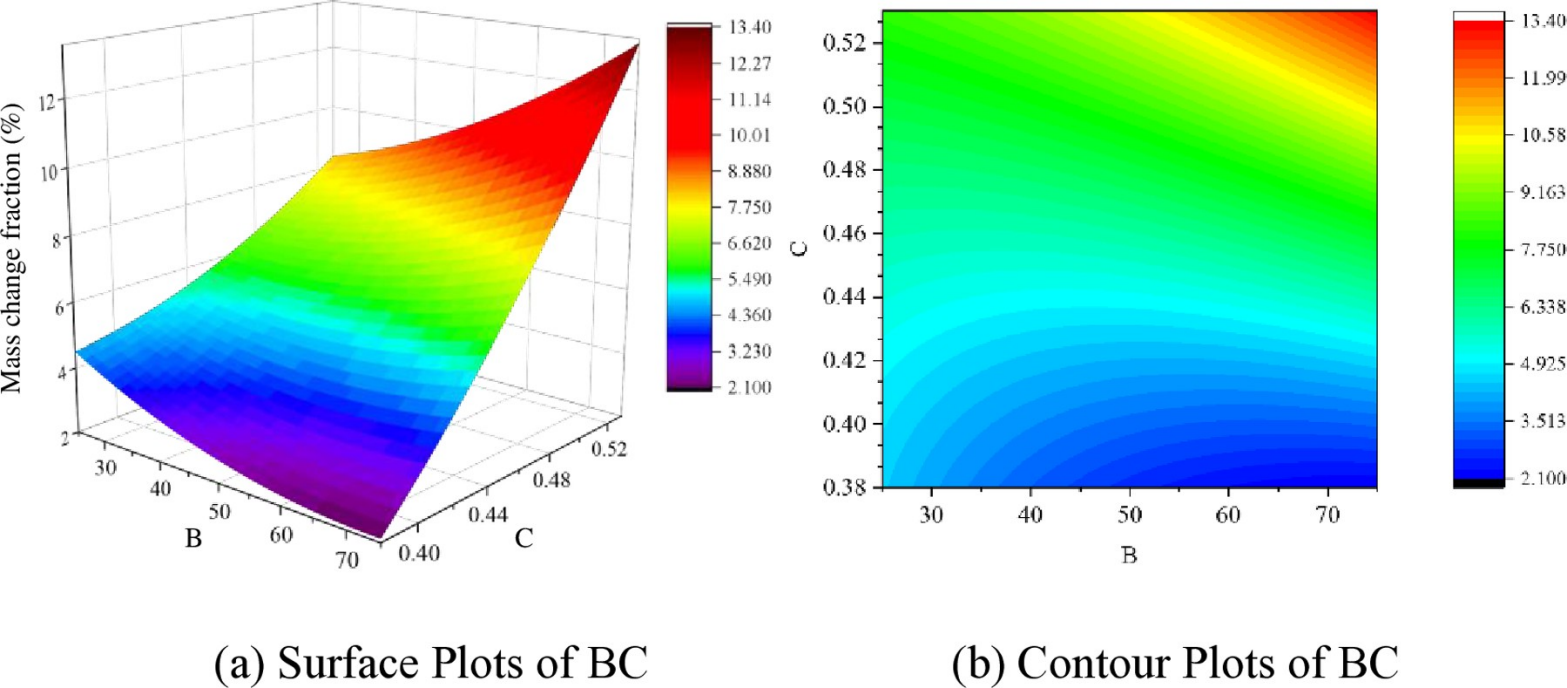
Response surface of BC factor interaction for 7 days of curing; (a) Surface Plots of BC; (b) Contour Plots of BC.

**Fig 13:**
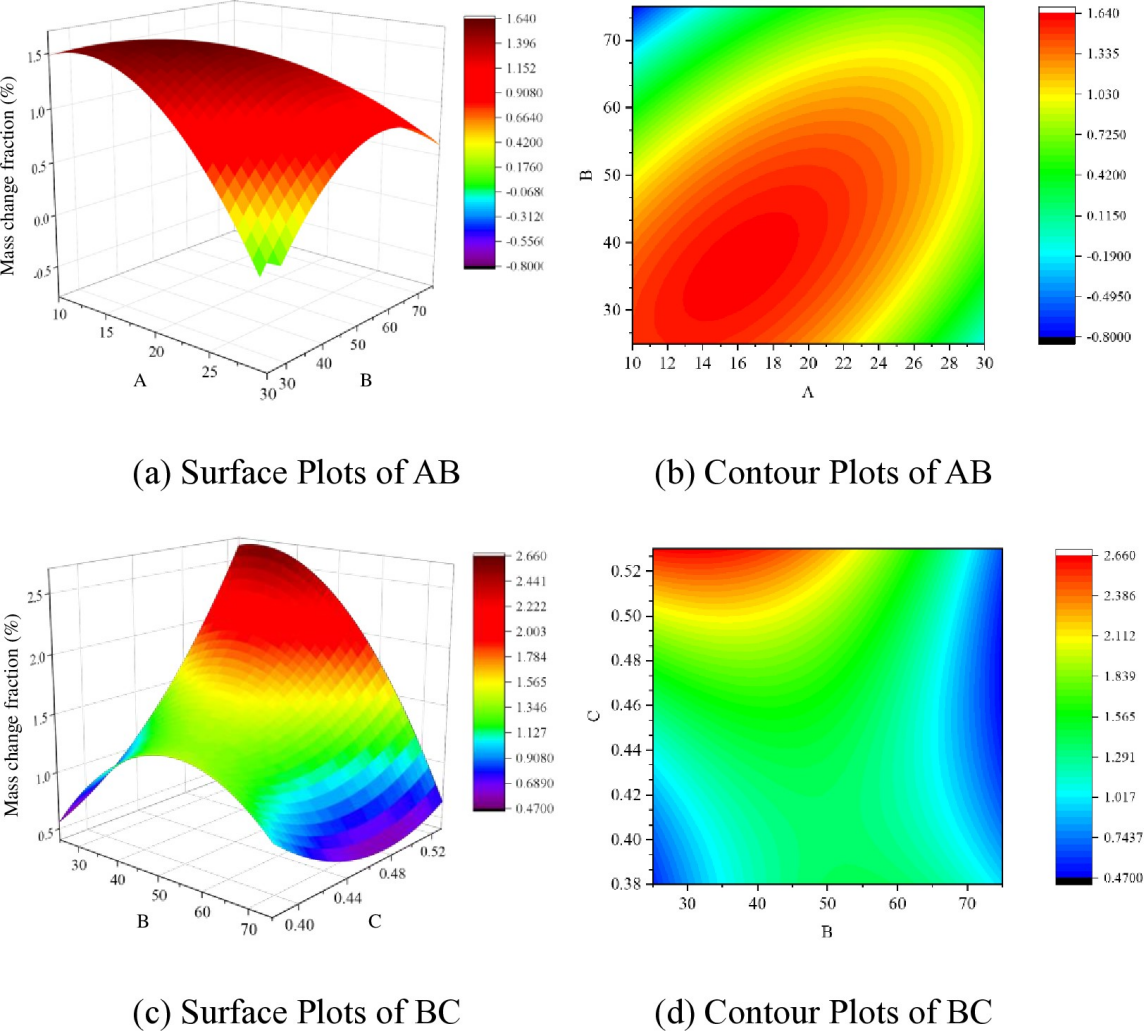
Response surface of BC factor interaction for 28 days of curing; (a) Surface Plots of AB; (b) Contour Plots of AB; (c) Surface Plots of BC; (d) Contour Plots of BC.

**Fig 14:**
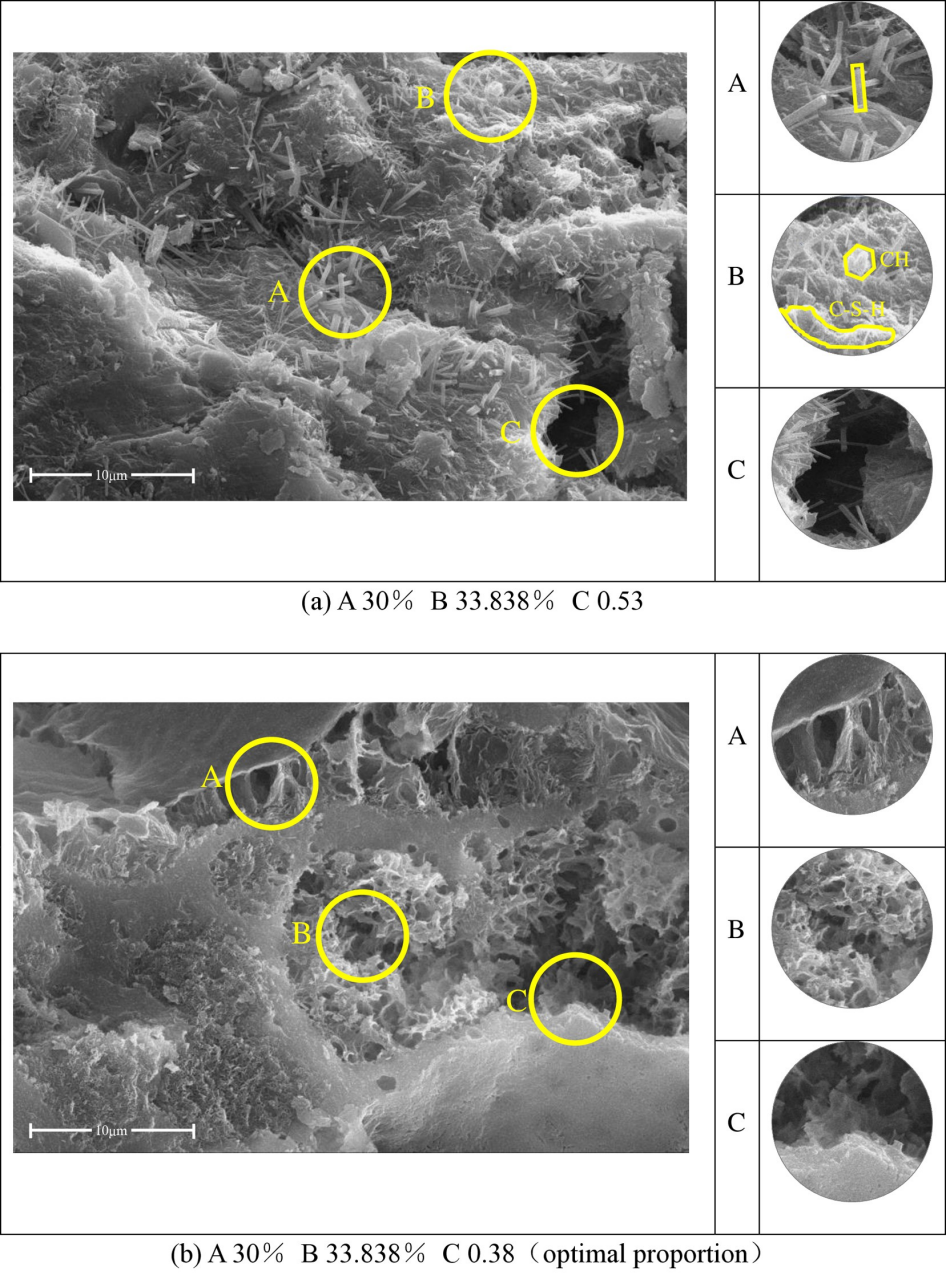
SEM diagram of sulfate erosion of substrate after curing for 7 days; (a) A 30% B 33.838% C 0.53; (b) A 30% B 33.838% C 0.38(optimal proportion).

The significant influencing factors for the 7-day, 28-day, and 56-day curing periods were analyzed based on Tables 8–10. Furthermore, the impact trends of these significant factors on the response values were analyzed through the establishment of 3D response surface plots (Figs 12–14).

From Fig 12(A), it can be observed that the mass loss after 24 cycles of wet-dry alternation and sulfate erosion decreases with the increase in the proportion of ceramic micro-powder in supplementary cementitious materials and the higher water-to-cement ratio. Concerning early strength, the impact curve of waste ceramic powder is gentler than that of the water-to-cement ratio, demonstrating that the water-to-cement ratio is a crucial indicator affecting the early-age matrix’s resistance to erosion. As shown in Table 8, the interaction effect of BC is significantly influential in the corrosion performance factors of the substrate after 7 days of curing. Supplementary cementitious materials behave differently from cement hydration processes, leading to variations in free water content due to differences in the proportion of supplementary cementitious materials. This difference is crucial for the significant impact of the BC interaction. Upon analysis, it is observed that sodium sulfate solution infiltrates the concrete pores and generates expansive erosion products inside the specimens. The crystallization pressure of these erosion products exceeding the tensile strength of the matrix material is a key factor causing internal structural deterioration. A higher water-to-cement ratio in the substrate and excessive free water result in more internal voids, enhancing sulfate permeability, increasing the extent of damage, and consequently, leading to more mass loss.

Table 9 reveals that the interaction effects of AB and BC are most pronounced in the substrate’s resistance to sulfate erosion after a 28-day curing period. Setting the water-to-cement ratio as the median, the impact lines of the AB and BC interactions are depicted in Fig 13. From Fig 13(A) and 13(C), it can be observed that with an increase in the dosage of supplementary cementitious materials, the rate of mass change of the substrate after 28 days of curing first increases and then decreases, reaching a maximum rate of change at around a 20% substitution rate, indicating the lowest erosion resistance. Similarly, as the content of ceramic waste powder increases, the erosion resistance of the substrate after 28 days of curing shows a trend of initially increasing and then decreasing, reaching the maximum rate of change at a 40% powder content, indicating the lowest erosion resistance. Additionally, with an increase in the water-to-cement ratio, the mass loss of the substrate after 28 days of curing initially decreases and then increases. There exists an optimal water-to-cement ratio within this range. Fig 13(C) and 13(D) demonstrate distinct elliptical curves within the contour lines of the response surface, indicating a highly significant AB and BC interaction. This can be attributed to the presence of a significant amount of hydrated calcium aluminate and calcium hydroxide in cement hydration products. The reaction of sulfates with these compounds leads to the formation of expansive ettringite and gypsum, causing substrate damage. Therefore, the judicious addition of ceramic waste powder to replace cement can reduce cement consumption, decrease the generation of corrosion products, and effectively lower the erosion mass.

In summary, under the conditions of wet-dry cycles and sulfate erosion, the water-cement ratio is the most significant factor affecting the resistance of the substrate to sulfate erosion during shorter curing periods. As the water-cement ratio increases, the mass variation rate tends to increase, while the SCMs do not fully exert their effect. The dosage of CWP has the most significant impact on the corrosion resistance of the substrate during normal curing periods. With an increase in the proportion of CWP, the mass variation rate exhibits a trend of initially increasing and then decreasing. However, for long-term cured substrates, the influence of CWP is not significant. On the other hand, as the substitution rate of FA increases, the mass variation rate shows a decreasing trend. This can be attributed to the earlier hydration of CWP compared to FA.

### 3.4 Mix ratio optimization

In the optimization of the performance of CWP cementitious materials under the conditions of wet-dry cycles and sulfate erosion, the substitution rate of the SCMs, the proportion of CWP, and the water-cement ratio are considered as the dependent variables. The objective of the optimization is to minimize the mass variation rate of the cementitious materials at 7-day, 28-day, and 56-day curing periods. By employing the response surface methodology, the response surface objective design table (Table 11) can be obtained for optimization purposes.

**Table 11:** Response surface target design.

Index	Goal	Lower Limit	Upper Limit
A	In range	10	30
B	In range	25	75
C	In range	0.38	0.53
F-7	minimize	2	13.04
F-28	minimize	-0.73	2.42
F-56	minimize	-2.33	0

The optimal design parameters for the mix proportion are as follows: the substitution rate of 30%, the CWP content of 33.838%, and the water-cement ratio of 0.38. The cementitious materials were prepared using the optimal mix proportion and subjected to wet-dry cycles and sulfate erosion under curing periods of 7 days, 28 days, and 56 days. The experimental results of the mass variation of the cementitious materials under 24 cycles of wet-dry and sulfate erosion conditions for CWP cementitious materials were compared and verified with the predicted values calculated by the model. The verification results are shown in Table 12.

**Table 12:** Response surface optimization value error.

Index	F-7	F-28	F-56
Predictive value (%)	4.545	-0.731	-1.856
True value (%)	4.35	-0.71	-1.73
Error (%)	-4.29	-2.87	-6.79

From Table 12, it can be observed that the errors between the predicted values and the actual values of the mass variation rates at F-7, F-28, and F-56 are within a small range of 2.8 to 6.8. This indicates a high reliability of using the regression model for predicting and optimizing the target parameters. The main effects of mineral admixtures in concrete include: 1) based on the particle morphology characteristics of mineral admixtures, the proportional addition of SCMs helps to achieve a more reasonable particle grading of the cementitious paste, resulting in a well-compacted structure of the cementitious materials and an improved resistance to sulfate erosion; 2) the incorporation of mineral admixtures contributes to the refinement of hydration products in the cementitious materials, leading to a reduction in the generation of Ca(OH)_2_ [12]; 3) the addition of mineral admixtures can refine and close the micropores in the microstructure of the cementitious materials, thereby enhancing their corrosion resistance performance [21].

## 4. Microstructural characterization

**Fig 15:**
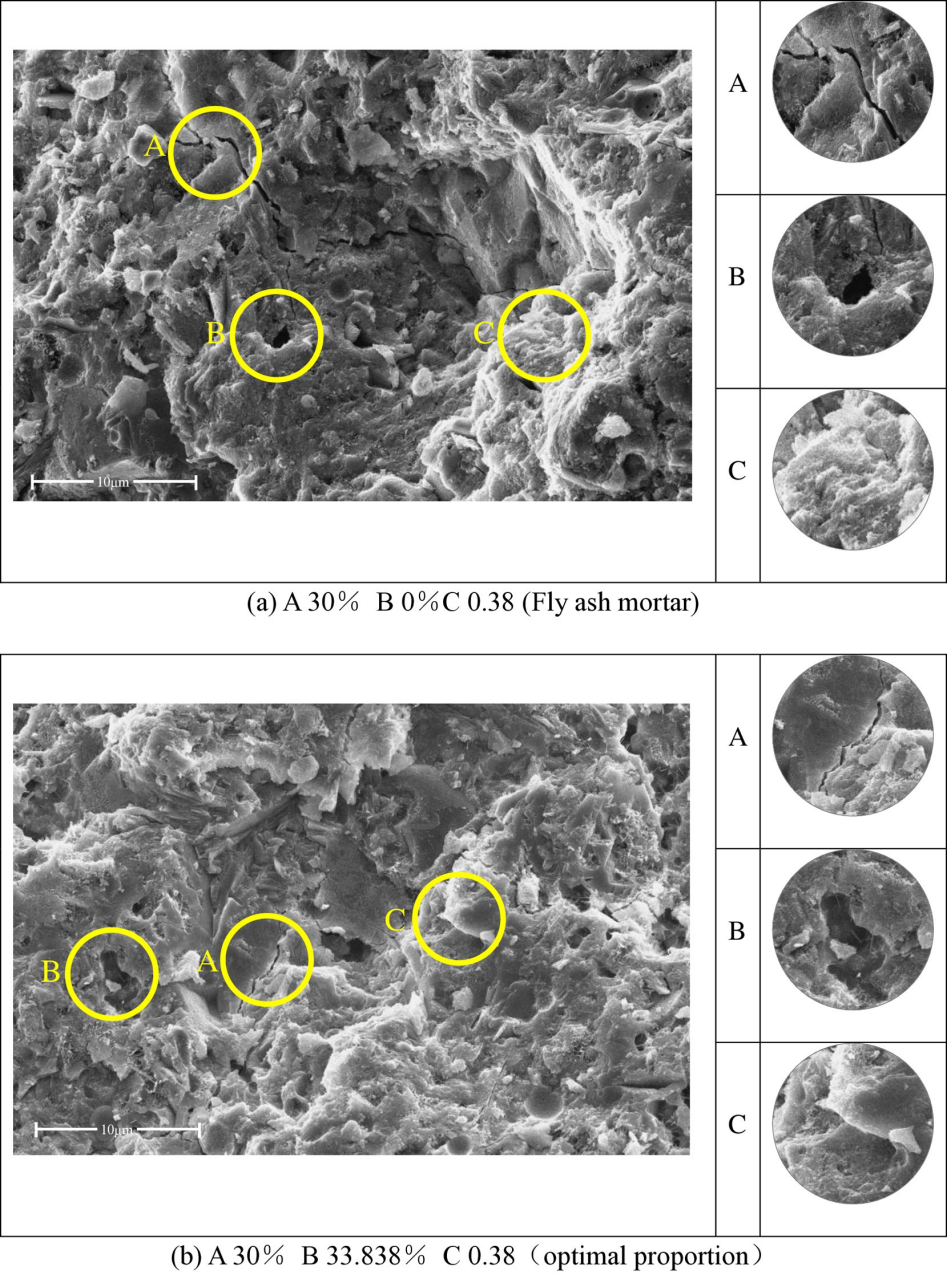
SEM diagram of sulfate erosion of substrate after curing for 28 days; (a) A 30% B 0%C 0.38 (Fly ash mortar); (b) A 30% B 33.838% C 0.38(optimal proportion).

**Fig 16:**
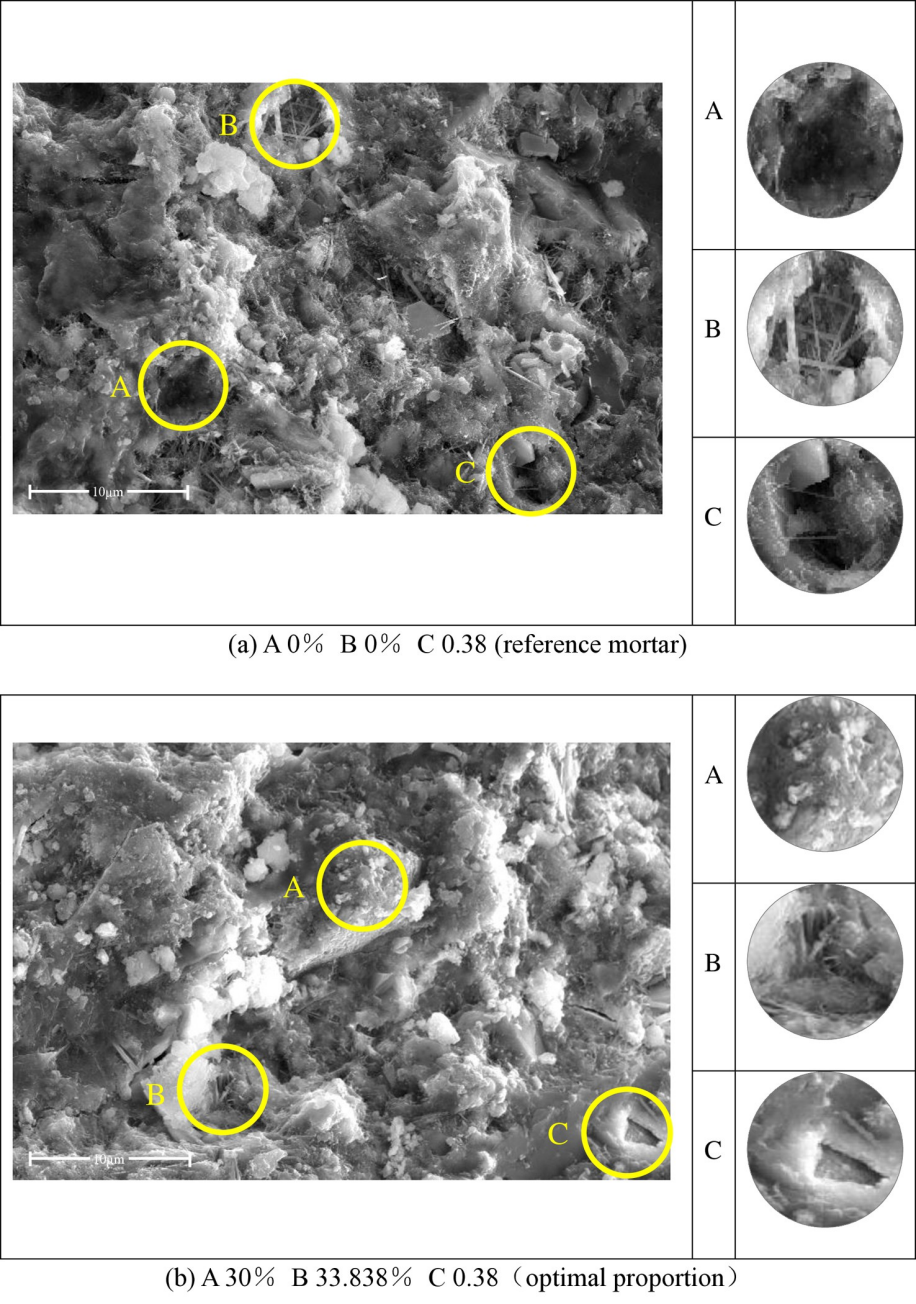
SEM diagram of sulfate erosion of substrate after curing for 56 days; (a) A 0% B 0% C 0.38 (reference mortar); (b) A 30% B 33.838% C 0.38(optimal proportion).

Although the chemical changes under the attack of Na_2_SO_4_ solution are not so complex, various changes occur at the microscopic level in the structure. Figs 14–16 are typical SEM images after time erosion with different mixture ratios. The comparative analysis of the typical corrosion morphologies was conducted on two sets of substrates with different water-cement ratios. Fig 14(A) illustrates the main hydration products, including ettringite (A), calcium silicate hydrate gel (B), and hexagonal calcium hydroxide (C).

Response surface analysis results indicate that the most significant factor affecting substrate corrosion after 7 days of curing is the water-to-cement ratio. Fig 14(A) represents a water-to-cement ratio of 0.53, while Fig 14(B) depicts the optimal mix with a water-to-cement ratio of 0.38. It is evident from the figures that early addition of supplementary cementitious materials delays hydration, serving merely as physical fillers in the structure. The corrosion pores are filled with ettringite. Lower water-to-cement ratios result in a smoother surface, with flocculent corrosion products and less ettringite observed. This demonstrates that appropriately reducing the water-to-cement ratio significantly impacts the early-age substrate’s corrosion resistance. This finding is consistent with the conclusions in references [50]. The potential reason for this could be the difference in porosity between sample 14(a) with a higher w/c ratio and sample 14(b) with a lower w/c ratio. Structures with higher w/c ratios exhibit greater porosity, with 14(a) showing evident large pores (C), making them more susceptible to ionic attacks. Sample 14(b), with a lower water-to-cement ratio, also presents corrosion-induced pores but filled with other corrosion products (C). The primary degradation mechanism in high water-to-cement ratios is gypsum crystallization corrosion, as evidenced by the presence of abundant needle-like ettringite in 14(a) (A). In contrast, the main degradation mechanism in low water-to-cement ratios is ettringite corrosion (A) in 14(b).

The most significant factors influencing the corrosion of the substrate at 28-day curing are the proportion of CWP and FA. The typical corrosion morphologies of substrates with different proportions of SCMs were selected for analysis. Fig 15(A) represents the case of single addition of 30% FA, while Fig 15(B) represents the case of simultaneous addition of 67% FA and 33% CWP In Fig 15(A), the corrosion image displays wider corrosion cracks (A) and deeper cavities (B). In Fig 15(B), the substrate with the addition of SCMs shows significantly shorter and finer cracks (A), with slightly larger but shallower cavities, which are filled with substances (B). This indicates that the corrosion resistance of the substrate with SCM addition is stronger than that of FA alone. Factors affecting the substrate’s resistance to sulfate erosion at 28-day curing include the proportion of CWP and the water-cement ratio. Analyzing the particle morphology of mineral admixtures, the specific surface area of CWP is larger than that of FA, leading to greater water absorption in the mixture and indirectly reducing the water-cement ratio. This enhances the substrate’s resistance to erosion.

Fig 16 presents the typical morphologies of the corroded substrates after 56-day curing as observed through SEM images. Fig 16(A) represents the reference cement-based substrate, while Fig 16(B) represents the optimal mix proportion of the substrate designed in this study using response surface methodology. Comparing Figs 14 and 15, it can be observed that the calcium silicate hydrate gel continues to increase and the hydration products become denser at hydration times of 7, 28, and 56 days. In Fig 14(B), during the 7-day hydration period, the formation of calcium silicate hydrate gel is incomplete, exhibiting flocculent distribution (B). The loose hydration products make it easier for sulfate ions to penetrate the substrate, resulting in significant deterioration in corrosion resistance. From Figs 15(A)(C), 15(B)(C), 16(A)(A) and 16(B)(A), it can be observed that with the continuous progress of cement hydration, the calcium hydroxide (Ca(OH)_2_) serves as the basis for the secondary hydration reaction of SCMs. The addition of SCMs significantly enhances the resistance to sulfate erosion from 28 to 56 days, and the generated calcium silicate hydrate gel effectively fills the voids and reduces the porosity [16]. The decrease in quality caused by the corrosion of the cementitious matrix becomes less pronounced as the curing age increases. This conclusion aligns with the results obtained from the response surface experiments.

Fig 16(A) exhibits deeper cavities (B) (C) after the corrosion of the plain paste, while Fig 16(B) shows evident corrosion traces with no formation of voids (B) (C) after the addition of SCMs. This can be attributed to the more reasonable particle size distribution of the supplementary cementitious materials. The addition of SCMs with finer particles can further fill the microscopic voids within the cementitious materials, thereby improving the overall density of the substrate. Consequently, it exhibits stronger resistance to erosion compared to plain paste [51, 52]. The microscopic morphology of the SCMs, as obtained through the morphology analysis in Section 2.2.1, reveals that the CWP has irregular polygons, while the FA possesses spherical particles. There is a synergistic effect between the two materials, and at an appropriate proportion of simultaneous addition, it can exhibit performance superior to either material alone.

**Fig 17:**
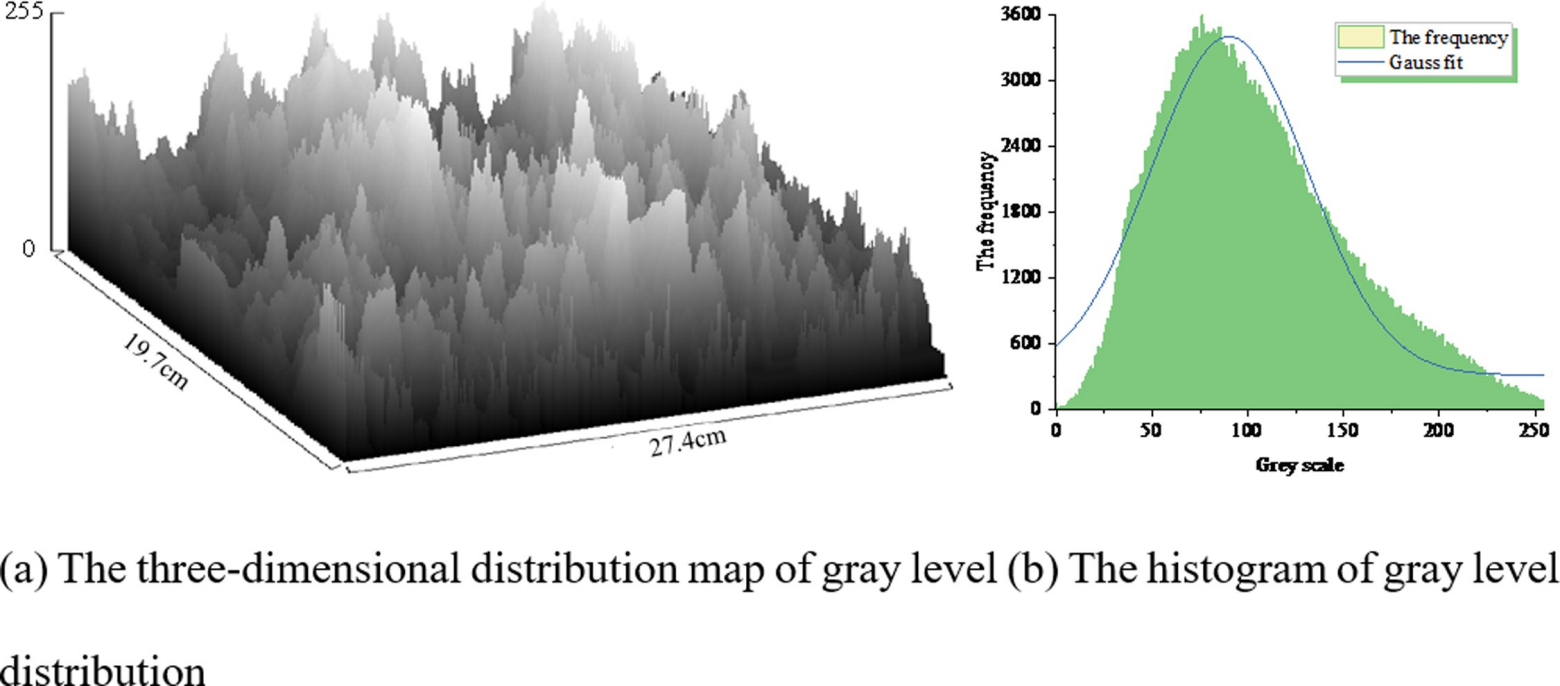
Grayscale distribution of SEM images of clean pulp; (a) The three-dimensional distribution map of gray level; (b) The histogram of gray level distribution.

**Fig 18:**
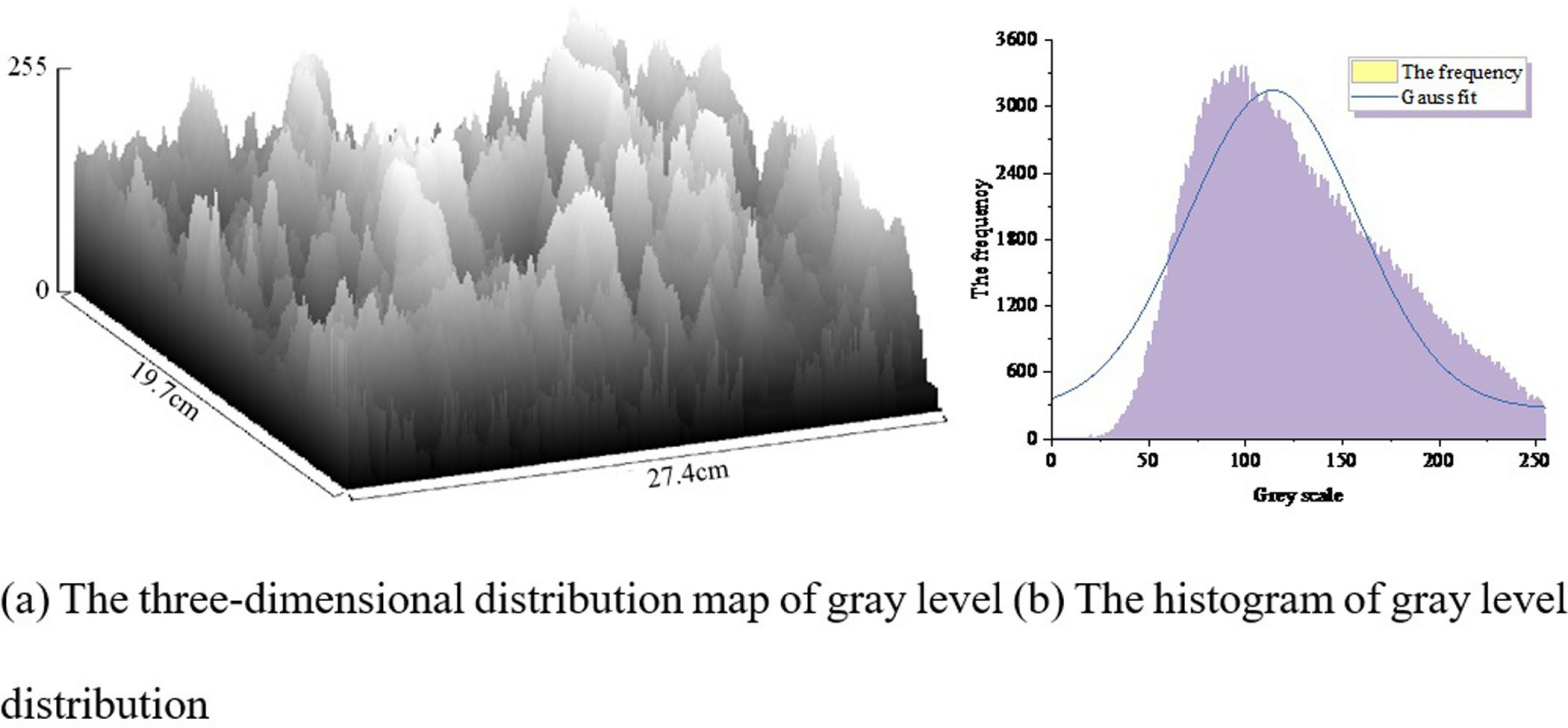
Grayscale distribution of SEM image of complex blended; (a) The three-dimensional distribution map of gray level; (b) The histogram of gray level distribution.

There is a distinction between the plain paste and the optimal density. To illustrate this, typical SEM images of the plain paste and the optimal density, namely Fig 15(A) and 15(B), were selected. The grayscale values of the images were used as the Z-axis on a scale of 0 to 255, while the length and width of the images were taken as the x and y axes, respectively. The three-dimensional distribution of grayscale is shown in Figs 17(A) and 18(A). The frequency of occurrence of each grayscale value was extracted as the y-axis, while the grayscale values were plotted on the x-axis to create histograms depicting the grayscale distribution, as shown in Figs 17(B) and 18(B).

Nonlinear fitting was performed on the histograms, as shown in Table 13. The mean values of Figs 17(A) and 18(A) are identical, ensuring comparability between the two images. The goodness of fit for Fig 17(B) is 0.936, while for Fig 18(B) it is 0.866, indicating a relatively high goodness of fit that supports a normal distribution. This suggests that the grayscale values of the selected pixels in the two SEM images are randomly distributed, reflecting good image quality.

**Table 13:** Gray-level histogram model of SEM images.

Index	Clear paste image	Compound SCMs image
**Model**	Gauss	
**Equation**	$y=y_{0}+\frac{A}{w\sqrt{\pi /2}}e^{-2\frac{{\left(x-x_{c}\right)}^{2}}{w^{2}}}$	
***y*_0_**	311±37	267±65
***x*_c_**	90±0.64	114±0.96
***w***	81±1.80	87±3.28
**A**	31023±8888	316234±16067
**Mean**	1394	1393
**Standard Deviation**	1172	1079
**Minimum**	312	284
**Maximum**	3398	3145
**R-Square (COD)**	0.936	0.866

Table 13 reveals that the grayscale difference in Fig 17(A) is 3086 for the neat slurry, while the optimal mix grayscale difference for the co-blended SCMs in Fig 18(A) is 2861. The variance of the grayscale for the neat slurry, 1172, is higher than the variance after co-blending, which is 1079. This indicates significant pixel discrepancies, suggesting greater roughness and lower density of the neat slurry material. This phenomenon can be attributed to the "gradient hydration effect" resulting from the blending of two supplementary cementitious materials with differing fineness and reactivity. As the curing age progresses, the hydration reaction rates of various materials at different stages vary, with each age period’s different cementitious materials playing a role in compensating for strength deficiencies and activating the hydration reaction of another admixture, thereby enhancing the substrate’s corrosion resistance. The inclusion of fly ash and ceramic waste powder reduces cement consumption, and the secondary hydration of mineral admixtures consumes a large amount of CH crystals, leading to a decrease in CH content in the cement paste, thus improving its resistance to sulfate erosion damage. Moreover, the substitution of some cement with mineral admixtures reduces the content of active aluminum phases in the cement paste, to some extent alleviating the formation of ettringite under sulfate attack, which could lead to deterioration.

## 5. Conclusions

Results of an experimental study investigating the effect of co-blending fly ash and ceramic waste powder on the sulfate erosion resistance of cement-based materials were presented. The experimental design utilized a Box-Behnken Design model within the response surface methodology, considering three key factors: the substitution of supplementary cementitious materials, the dosage of ceramic waste powder, and the water-to-cement ratio. The study aimed to establish a response surface model to analyze the significance of individual factors and their interactions on the experimental outcomes. Macroscopic erosion images and SEM micrographs were quantitatively analyzed to elucidate the mechanisms by which co-blending supplementary cementitious materials enhances the durability of cement-based materials. The conclusions drawn are as follows:

1. The addition of 20% fly ash improves the compressive strength of cement-based materials, while the compressive strength decreases with increasing ceramic waste powder content when used alone. Under wet-dry cycling conditions, the resistance of ceramic waste powder cement-based materials to sulfate corrosion surpasses that of fly ash-based materials.
2. A response surface model for the mass loss rate of cement-based materials after sulfate corrosion under wet-dry cycling conditions was established. Variance analysis of the regression model indicated that all model P-values were below 0.05, demonstrating high reliability and fitting accuracy of the model. This model can be employed to predict actual test results.
3. Significance analysis of single factors on the rate of substrate mass change after corrosion revealed that under short-term curing conditions (7 days), the water-to-cement ratio is the most significant factor leading to mass changes in the substrate after corrosion. Apart from the water-to-cement ratio, the dosage of ceramic waste powder is also an important influencing factor on the corrosion performance of cement-based materials under normal curing conditions (28 days). Following long-term curing (56 days), the corrosion resistance of cement-based materials is primarily determined by the content of supplementary cementitious materials.
4. 3D response surface analysis of multifactor interactions revealed that the substitution rate and proportion of different types of supplementary cementitious materials are the most crucial factors affecting the corrosion resistance of substrates. The interaction between the water-to-cement ratio and supplementary cementitious materials also significantly impacts the resistance to sulfate erosion. Optimal substrate resistance to wet-dry cycling sulfate erosion is achieved when 30% of the cement is replaced by supplementary cementitious materials, ceramic waste powder accounts for 33.838% of SCMs, and the water-to-cement ratio is 0.38.
5. Comparative analysis of SEM microscopic grayscale three-dimensional distribution maps and histograms showed a net slurry grayscale difference of 3086 and an optimal mix grayscale difference of 2861 after co-blending SCMs, indicating an increase in matrix density after the co-blending of SCMs. This serves as crucial evidence for the enhancement of substrate durability.
